# Epitaxially Integrated Electro‐Optic KNbO_3_ Thin Films on Silicon

**DOI:** 10.1002/adma.75067

**Published:** 2026-09-28

**Authors:** Sankalpa Hazra, Tobias Schwaigert, Yingxin Zhu, Aiden Ross, Muhammad Aminul Haque Chowdhury, Shun‐Li Shang, Sameer Singh, Zhiyu Zhang, Zhe Wang, Brynn Brower, Saugata Sarker, Suchismita Sarker, Zi‐Kui Liu, Rama K. Vasudevan, Long‐Qing Chen, Yang Yang, Darrell G. Schlom, Venkatraman Gopalan

**Affiliations:** ^1^ Department of Materials Science and Engineering The Pennsylvania State University University Park Pennsylvania USA; ^2^ Platform For the Accelerated Realization Analysis, and Discovery of Interface Materials (PARADIM) Cornell University Ithaca New York USA; ^3^ Department of Materials Science and Engineering Cornell University Ithaca New York USA; ^4^ Department of Engineering Science and Mechanics The Pennsylvania State University University Park Pennsylvania USA; ^5^ Department of Electrical Engineering The Pennsylvania State University University Park Pennsylvania USA; ^6^ Department of Physics The Pennsylvania State University University Park Pennsylvania USA; ^7^ Cornell High Energy Synchrotron Source Cornell University Ithaca New York USA; ^8^ Center For Nanophase Materials Sciences Oak Ridge National Laboratory Oak Ridge Tennessee USA; ^9^ Kavli Institute at Cornell for Nanoscale Science Ithaca New York USA; ^10^ Leibniz‐Institut Für Kristallzüchtung Berlin Germany

**Keywords:** epitaxy, ferroelectricity, materials science, optoelectronics, photonics, pockels effect, silicon, silicon photonics, thin film, transmission electron microscopy

## Abstract

The absence of a native electro‐optic effect in silicon remains a fundamental limitation for integrated photonics, motivating the integration of ferroelectric oxides as active materials. Here, we report the first epitaxial integration of KNbO_3_ thin films on silicon, establishing a new materials platform for silicon‐integrated electro‐optics. Silicon integration of KNbO_3_ has remained unexplored due to potassium volatility challenges during synthesis. Employing suboxide molecular‐beam sources assisted by in situ RHEED monitoring and guided by thermodynamic simulations, we establish an adsorption‐controlled growth window for phase‐pure KNbO_3_ films. Temperature dependent x‐ray diffraction, optical second‐harmonic generation, piezo‐response force microscopy, and transmission electron microscopy aided by phase‐field simulations confirm high‐quality epitaxial films with sharp interfaces and structural phase transitions mirroring KNbO_3_ single crystals. Electro‐optic measurements demonstrate a linear Pockels response with an effective electro‐optic response of 146  ±  14 pm V^−1^ at 1550 nm. Phase‐field simulations further predict that modest compressive strain can drive the electro‐optic response to ∼900 pm V^−1^. Beyond KNbO_3_, this integration pathway provides access to the broader (K,Na)(Ta,Nb)O_3_ family, opening pathways to harness their strong electro‐optic response, nonlinear optical response, piezoelectric response and ferroelectric properties for next‐generation silicon photonics and electronics beyond current BaTiO_3_‐based approaches.

## Graphical abstract

We demonstrate the first epitaxial integration of electro‐optic KNbO_3_ thin films on silicon using suboxide molecular‐beam epitaxy. Combining adsorption‐controlled molecular‐beam epitaxy, advanced characterization, and phase‐field modeling, we demonstrate phase‐pure crystalline films mirroring bulk crystals with robust Pockels response, enabling future silicon‐integrated electro‐optic, nonlinear photonic, and piezo‐optomechanical technologies.

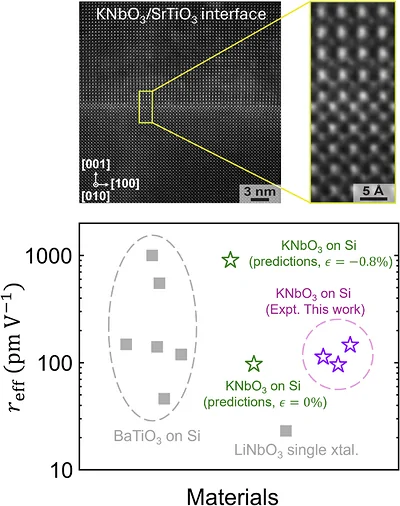

## 1 Introduction

The integration of functional oxides with silicon has emerged as a critical pathway toward overcoming fundamental limitations in photonics and electronics [1, 2]. Silicon, despite its dominance in microelectronics and increasing role in photonics, lacks useful properties that are present in ferroelectrics such as the electro‐optic effect. This constrains the development of key functionalities including efficient electro‐optic modulation [3] and on‐chip optical switching [4]. Ferroelectric perovskite oxides offer a solution by providing strong electro‐optic effects and large spontaneous polarization. Integration of these oxides directly onto silicon substrates enables monolithic devices that leverage silicon's mature processing infrastructure, low cost, and CMOS compatibility while accessing the functional capabilities of complex oxides. The successful epitaxial integration of ferroelectric perovskites on silicon has validated this approach. BaTiO_3_ has been demonstrated as a platform for electro‐optic modulators in silicon photonics, exploiting its Pockels response [2].

Among oxide ferroelectrics, potassium niobate (KNbO_3_) presents a compelling case for silicon integration. KNbO_3_ possesses a polar‐optic coefficient, *g*_1111_, that is 50% larger [5] than that of BaTiO_3_, reflecting a stronger intrinsic coupling between ferroelectric polarization and optical properties, and exhibits bulk electro‐optic coefficients of *r*_42_ = 450  ±  30 pm V^−1^  [6]. A *T*_c_ of 676 K [7] in bulk KNbO_3_ provides a wide operational window over which its electro‐optic performance can be sustained, exceeding that of bulk BaTiO_3_ [8]. Additionally, KNbO_3_ has superior transverse electrostrictive coefficient (|*Q*_13_|  ≈  0.053 m^4^C^−2^) [8], significantly larger than that of BaTiO_3_ (|*Q*_13_|  ≈  0.034 m^4^C^−2^) [8, 9] and PbTiO_3_ (|*Q*_13_|  ≈  0.026 m^4^C^−2^) [8, 10], providing exceptionally strong coupling between strain and ferroelectric polarization. This strong strain‐polarization coupling translates directly into enhanced piezoelectric coefficients [11], opening pathways toward high‐performance piezo‐optomechanical photonic transducers on silicon [12].

Beyond these intrinsic properties, KNbO_3_ serves as the entry point to the (K,Na)(Ta,Nb)O_3_ family on silicon, a compositional space whose functional potential for photonics and electronics remains largely unexplored. For example, KNbO_3_ [8, 13] and (K,Na)NbO_3_ [14, 15] are already known to produce large enhancements in nonlinear optical properties. Additionally, K(Ta,Nb)O_3_ which is accessible through the same compositional space, exhibits a giant quadratic electro‐optic response [16], enabling applications in beam steering and photorefractive devices inaccessible to conventional Pockels materials.

Despite these attributes, synthesis challenges arising from potassium volatility have limited efforts to integrate KNbO_3_ on silicon substrates. Based on our recent demonstration of the epitaxial growth of KNbO_3_ thin films by suboxide molecular‐beam epitaxy (*S*‐MBE) on rare‐earth scandate substrates (ReScO_3_, Re = Gd and Dy) [8], in this work we extend this epitaxial integration to silicon using a SrTiO_3_ buffer layer. We employ transmission electron microscopy and synchrotron x‐ray diffraction to characterize the crystallinity and epitaxial quality of the strain‐relaxed KNbO_3_/SrTiO_3_/Si heterostructures. Temperature‐dependent optical second‐harmonic generation, x‐ray diffraction, and piezo‐response force microscopy aided by phase‐field simulations is used to probe phase transitions and domain structures. Finally, electro‐optic measurements are performed to determine the room temperature electro‐optic coefficient of KNbO_3_ integrated on silicon. These results establish a viable pathway for incorporating this high‐performance, lead‐free ferroelectric into silicon photonic and electronic platforms.

### 1.1 Growth of KNbO_3_ Using Molecular‐Beam Epitaxy

Aided by thermodynamic calculations, we systematically map the adsorption‐controlled growth window of phase‐pure KNbO_3_ thin films on single‐crystal SrTiO_3_ substrates. This mapping provides clear guidance for the subsequent synthesis of KNbO_3_ on silicon substrates upon which a 100 nm SrTiO_3_ buffer layer is first deposited by MBE [17]. A thermodynamic framework (see Methods, Figure S1) was employed to evaluate the phase stability of potassium–niobium oxides as a function of potassium partial pressure and substrate temperature. These calculations are summarized in an Ellingham‐type phase stability diagram (Figure 1a), constructed at a fixed oxygen partial pressure of 7.5 × 10^−4^ Torr. This diagram serves as a practical guide for identifying regimes in which KNbO_3_ is thermodynamically favored over competing phases.

**FIGURE 1:**
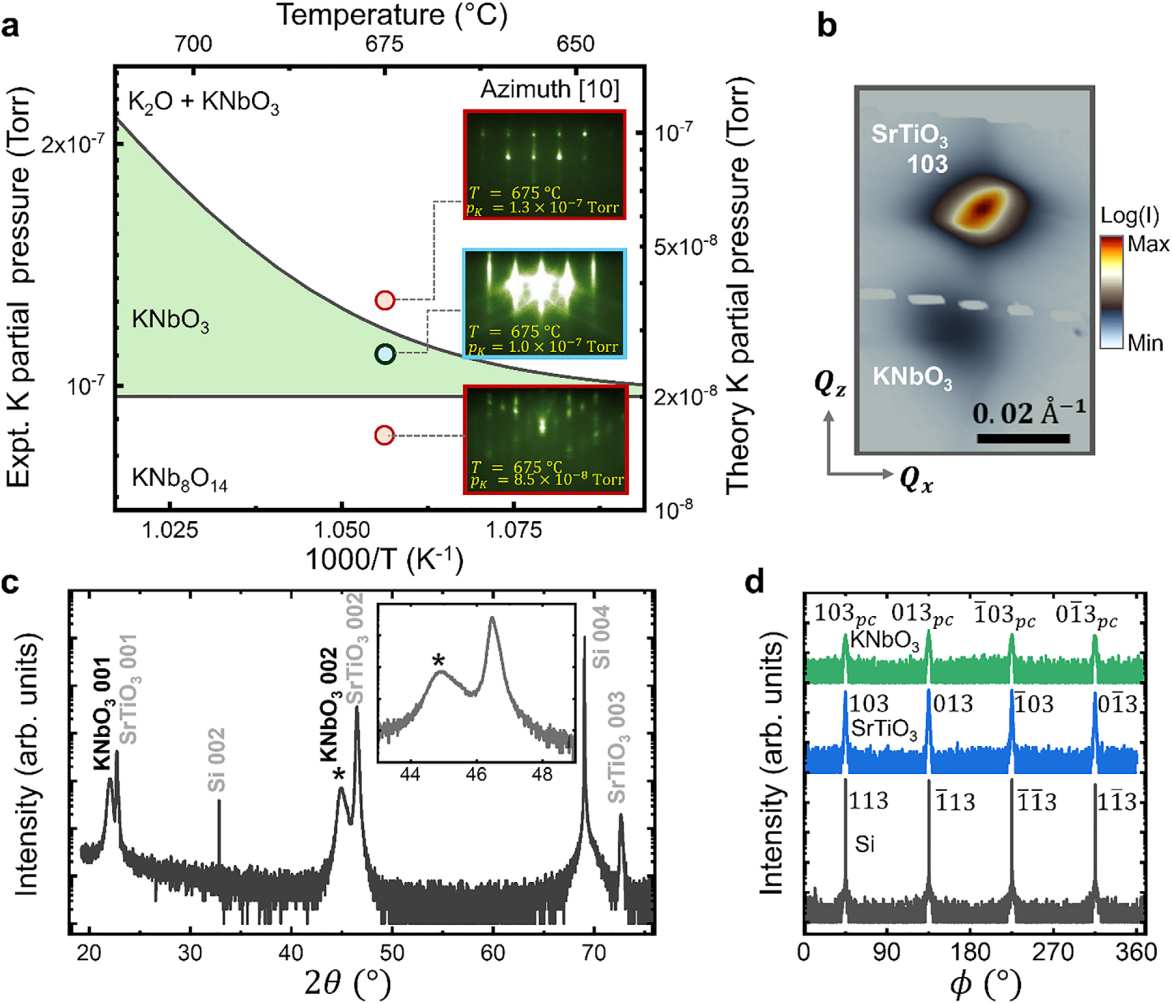
(a) Growth window for KNbO_3_: Theoretically calculated potassium partial pressure (right axis) as a function of growth temperature showing predicted adsorption‐controlled growth window for KNbO_3_ (shaded region), bounded by potassium‐rich (K_2_O + KNbO_3_) and potassium‐deficient (KNb_8_O_14_) phase conditions. Thermodynamically predicted potassium overpressures (Figure S1) are converted into potassium partial pressures required for the growth of KNbO_3_ (right axis) by adding the potassium partial pressure consumed during KNbO_3_ growth. Experimental potassium partial pressures, determined from QCM‐calibrated fluxes prior to each deposition, are shown on the left axis. In situ RHEED patterns for different growth regimes are shown in insets. The blue inset represents best experimental growth conditions while the red insets represent the potassium‐rich and potassium‐deficient conditions. Although the left and right (theory/experiment) axes are in qualitative agreement regarding the location of the adsorption‐controlled growth window for single‐phase KNbO_3_ (the green shaded region), quantitatively they do not precisely coincide. This is to be expected given the uncertainty of the thermodynamic parameters used and oxygen partial pressure. (b) Synchrotron XRD reciprocal space map around the 103 SrTiO_3_ and KNbO_3_ peaks. (c) Lab θ − 2θ XRD scans showing only 00ℓ peaks of Si, SrTiO_3_, and KNbO_3_. Inset shows zoom in around the 002 KNbO_3_ peak. (d) Lab XRD ϕ‐scans around KNbO_3_ 103_pc_, SrTiO_3_ 103 and Si 113‐type reflections.

To enable the growth of KNbO_3_ thin films, several approaches have been developed to address the synthesis challenges associated with highly volatile alkali‐containing oxides. One approach is the pulsed‐laser deposition (PLD) scheme, in which K_2_O is thermally evaporated [18]. In such a scheme, the gas‐phase species generated during K_2_O evaporation depend sensitively on the oxygen partial pressure, complicating quantitative control over the potassium chemical potential at the growth surface. In contrast, our approach relies on the direct evaporation of elemental potassium from an intermetallic KIn_4_ source, for which the vapor pressure of potassium exceeds that of indium by approximately eleven orders of magnitude [19]. This enables a stable and well‐defined potassium flux without introducing additional oxygen‐dependent gas‐phase equilibria. During growth, reflection high‐energy electron diffraction (RHEED) was recorded continuously, providing real‐time feedback on surface structure and phase evolution. Across the explored parameter space, multiple phases were observed. To directly probe phase stability and surface stoichiometry, we combine in situ RHEED with potassium partial pressures calculated from QCM‐measured K_2_O fluxes immediately prior to each growth (see Supporting Information).

Figure 1a summarizes the experimentally accessible growth regimes at 675°C as a function of potassium partial pressure. While in theory the growth window extends to low overpressures of potassium over a substrate surface (Figure S1), the range of potassium overpressures no longer provide a reasonable film growth rate below $∼\;\;10^{-8}$ Torr. For example, a substrate temperature of 675°C and a potassium overpressure of 10^−9^ Torr corresponds to a potassium flux of $∼\;\;7\times 10^{11}$ K atoms cm^−2^ s^−1^, [20], which if it were all consumed to form KNbO_3_ would yield a KNbO_3_ growth rate of just 0.005 Ås^−1^. To compare the theoretical overpressure of potassium needed to stabilize KNbO_3_ to the experimental potassium partial pressures (*p*_K_) used for the growth of KNbO_3_, one would need to add the potassium partial pressure corresponding to the potassium flux that is being consumed to form KNbO_3_ (which is equal to the niobium flux used for KNbO_3_ growth) to the theoretically predicted potassium overpressures.

Experimentally, typical potassium fluxes used for growth range from $∼\;\;6.0\;\times 10^{13}$ to 9.2  ×  10^13^ atoms cm^−2^ s^−1^ with a Nb flux of $∼\;\;1.5\;\times 10^{13}$ atoms cm^−2^ s^−1^. To better compare our thermodynamic calculations of the potassium overpressure conditions under which KNbO_3_ is stable with the experimental conditions under which we have grown KNbO_3_, we construct a theoretical potassium partial pressure axis in Figure 1a. In this axis, the partial pressure of potassium being consumed during growth is added to the overpressure needed according to our thermodynamic calculations. In our case, with a NbO_2_ flux of $∼\;\;1.5\times 10^{13}$ atoms cm^−2^ s^−1^, the flux of potassium being consumed during growth is also $∼\;\;1.5\times 10^{13}$ atoms cm^−2^ s^−1^. The corresponding potassium partial pressure $∼\;\;2\times 10^{-8}$ Torr then compares to the theoretical case of having no potassium overpressure.

Experimental potassium overpressures (*p*_K_) achieved during growth are calculated to range from 8.5  ×  10^−8^ Torr to 1.3  ×  10^−7^ Torr corresponding to potassium fluxes ranging from 6.0  × 10^13^ to 9.2  ×  10^13^ atoms cm^−2^ s^−1^. At high potassium overpressures (e.g., the potassium‐rich condition, *p*_K_  ≈  1.3  ×  10^−7^ Torr), a spotty RHEED pattern is observed, indicating higher surface roughness owing to excess potassium accumulation at the surface, consistent with the coexistence of K_2_O and KNbO_3_ predicted from thermodynamic calculations under potassium‐rich conditions. In contrast, at an intermediate potassium overpressure of *p*_K_  ≈  1.1  ×  10^−7^ Torr (ideal condition), sharp and well‐defined streaky RHEED features are observed, characteristic of smooth, two‐dimensional growth, and phase‐pure KNbO_3_. Reducing the potassium overpressure further to *p*_K_ ≈  8.5  ×  10^−8^ Torr (a potassium deficient condition) results in a loss of streak intensity, increased diffuse scattering, and additional diffractions patterns signaling the onset of competing niobium‐rich phases, consistent with thermodynamic predictions identifying the formation of KNb_8_O_14_.

Employing in situ RHEED monitoring assisted by thermodynamic simulations provides a roadmap that enables immediate correction of growth parameters. Deviations from the optimal growth window can be detected in real time and compensated by adjusting the potassium flux or substrate temperature, allowing the system to be steered back into the adsorption‐controlled regime highlighted in Figure 1a. Additionally, using a similar growth strategy, layer‐by‐layer growth of KNbO_3_ has previously been demonstrated on closely lattice‐matched ReScO_3_ substrates. When KNbO_3_ is grown on SrTiO_3_, however, which imposes a larger lattice mismatch (∼2.7%) and exceeds the estimated critical thickness for strain relaxation (∼11−12nm), island growth can become energetically favored over layer‐by‐layer growth, potentially promoting a three‐dimensional growth mode. With a real‐time feedback loop established to assess the phase stability of KNbO_3_ growth on SrTiO_3_ single crystals, we next turn to the synthesis of KNbO_3_ on SrTiO_3_/Si. The synthesis of 100 nm SrTiO_3_ (001) on Si (001) is reported elsewhere [17]. RHEED patterns of KNbO_3_ on SrTiO_3_/Si confirming phase‐pure KNbO_3_ growth is shown in Figure S2.

To calculate the lattice parameter and strain state of KNbO_3_, synchrotron‐based reciprocal space mapping (RSM) was performed around the SrTiO_3_ 103 peak (Figure 1b). From the observed KNbO_3_ 103_pc_ peak, the average in‐plane and out‐of‐plane lattice parameters are found to be 3.99 ± 0.01 and 4.03  ± 0.01 Å, respectively (Subscript ‘*pc*’ stands for pseudocubic). This closely matches the expected room temperature orthorhombic phase lattice parameters of single crystal KNbO_3_ (*apc* = 3.97 Å,  *cpc* = 4.03 Å) [21], implying the strain‐relaxed nature of our films. Additionally, phase‐field simulations (see Supporting Information) of a KNbO_3_ thin film on SrTiO_3_/Si under 0% misfit strain reveal average lattice parameters of *apc* = 4.01 Å,  *cpc* = 4.02 Å at 300 K, consistent with our experimental measurements.

Lab x‐ray diffraction measurements were performed on KNbO_3_ on SrTiO_3_/Si to confirm the crystallinity and epitaxial relationship between KNbO_3_ film and the SrTiO_3_ buffer layer. The θ − 2θ x‐ray diffraction scan shown in Figure 1c exhibits only reflections corresponding to KNbO_3_ and the underlying SrTiO_3_/Si heterostructure, with no detectable secondary phases, confirming phase‐pure integration of KNbO_3_ on silicon. Additionally, rocking curve measurements around the KNbO_3_ 001 reflection reveal two components: a sharp central peak (FWHM = 0.0097°) superimposed on a broader component (FWHM = 0.7497°), reflecting mosaic spread of the film arising from threading dislocations and misoriented sub‐domains formed due to strain relaxation (Figure S2).

The in‐plane orientation relationship between the layers of the film and substrate was determined from off‐specular ϕ‐scans of KNbO_3_ 103_pc_, SrTiO_3_ 103 and Si 113 reflections. As shown in Figure 1d, the epitaxial orientation relationship is KNbO_3_ [100]_pc_ || SrTiO_3_ [100] || Si [110] confirming epitaxial growth of KNbO_3_ on SrTiO_3_/Si. The alignment between SrTiO_3_ [100] and Si [110] confirms the well‐known 45° rotation of the SrTiO_3_ unit cell with respect to the silicon conventional unit cell arising from a better lattice parameter match of SrTiO_3_ (*a*_[100]_ =  3.905 Å) along the Si [110] direction (*a*_[110]_/2 = 3.84 Å) [22]. The epitaxial strain‐relaxed nature of the KNbO_3_ thin film is further confirmed by large‐area RSM maps along both *Q_x_* and *Q_y_* directions, which show corresponding KNbO_3_ peaks aligned with every SrTiO_3_ peak (Figure S3).

AFM reveals a RMS surface roughness of 1.47 nm for a 18 nm thick film and 6.83 nm for a 96 nm thick film across a 10 µm × 10 µm area (Figure S4). A larger surface roughness would contribute to larger optical losses through scattering in waveguide geometries and hence presents a challenge for silicon integration. Roughness of the starting SrTiO_3_/Si base film (0.97 nm) and the strain relaxed nature of the KNbO_3_ films can contribute to the surface roughness. In comparison, surface roughness of ∼0.55 nm was previously measured in commensurately strained 22 nm thick KNbO_3_ films on closely lattice‐matched GdScO_3_ single crystal substrates using the same growth method [8]. These low roughness values can be achieved on silicon through lattice parameter tuned buffer layers replacing SrTiO_3_. For example, by varying the barium concentration of compositionally graded (Ba,Sr)TiO_3_ [23, 24] buffer layers, the lattice parameter can be tuned to eliminate or reduce the mismatch between the KNbO_3_ film and the buffer layer. Alternatively, compositionally graded SrSn_x_Ti_1‐x_O_3_ or SrGe_x_Ti_1‐x_O_3_ buffer layers can also be used to achieve a similar lattice parameter tuning [25, 26]. A closely lattice‐matched buffer layer would enable thicker commensurately strained KNbO_3_ thin films with surface roughness mimicking those of KNbO_3_ on GdScO_3_ films.

### 1.2 Structural Characterization by Transmission Electron Microscopy

To visualize the interface quality and crystalline structure of our films, transmission electron microscopy measurements were performed on KNbO_3_/SrTiO_3_/Si heterostructures. Figure 2a shows cross‐sectional high‐angle annular dark‐field (HAADF) images and corresponding elemental energy‐dispersive x‐ray (EDX) maps of an 18 nm thick KNbO_3_ thin film grown on SrTiO_3_/Si. More comprehensive HAADF‐STEM and EDX datasets for the two crystallographic viewing directions are shown in Figures S5 and S6, corresponding to the SrTiO_3_ $\left[1\bar{1}0\right]$ and [010] projections, respectively. From the EDX maps, a SiO_x_ region is visible between the silicon substrate and SrTiO_3_ buffer layer; it is created due to silicon oxidation owing to the high MBE growth temperatures which increases oxygen diffusion across the SrTiO_3_ layer [27]. The thickness of the SiO_x_ layer depends on growth parameters such as temperature and oxygen pressure, with previous reports demonstrating that even complete conversion of the silicon layer to SiO_x_ is possible under appropriate conditions [28]. Additionally, the high growth temperatures also promote silicon diffusion into the SrTiO_3_ layer, as observed from the elemental maps, which can lead to amorphization of SrTiO_3_ starting from the SrTiO_3_/SiO_x_ interface [29]. Silicon, however, does not penetrate the KNbO_3_ layer. These signify the importance of using a sufficiently thick SrTiO_3_ buffer layer for integration of oxide films on silicon. In this study, a ∼100 nm thick SrTiO_3_ buffer was used. The elemental maps otherwise reveal a sharp interface between the KNbO_3_ film and SrTiO_3_ buffer.

**FIGURE 2:**
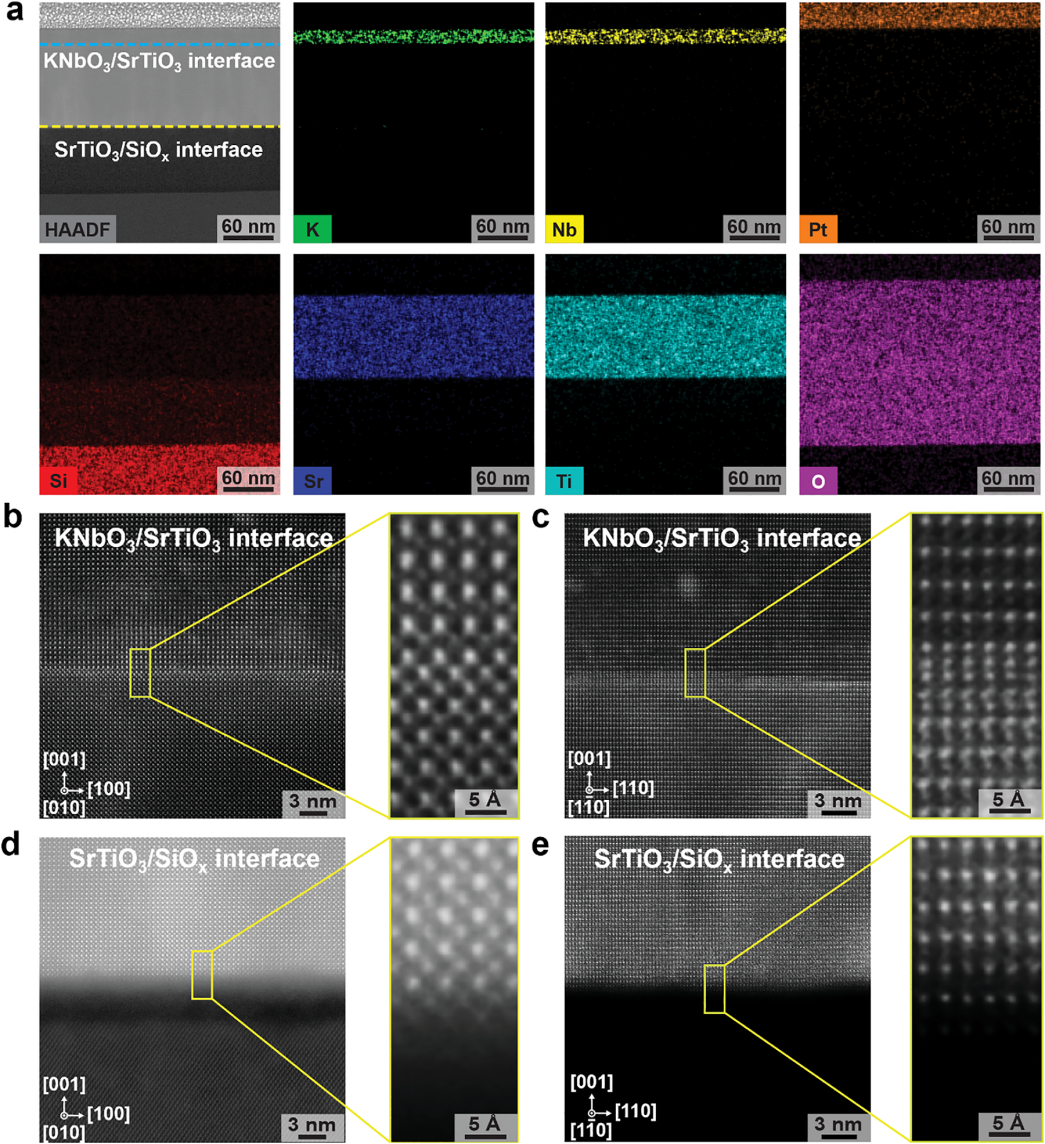
(a) Cross‐sectional scanning transmission electron microscopy (STEM) high‐angle annular dark‐field (HAADF) image of the KNbO_3_/SrTiO_3_/Si stack. The accompanying energy‐dispersive x‐ray spectroscopy (EDS) elemental maps (K, Nb, Sr, Ti, O, Si, and Pt) are acquired from the same field of view and illustrate the elemental distributions across the heterostructure. An amorphous SiO*_x_* interlayer is present between SrTiO_3_ and the Si substrate. (b,c) Higher‐magnification HAADF‐STEM images of the KNbO_3_/SrTiO_3_ interface viewed along the SrTiO_3_ [010] and [1$\bar{1}$0] zone axes, respectively. Insets show resolved atomic‐column arrangements consistant with local epitaxial alignment across the KNbO_3_/SrTiO_3_ interface. (d,e) Higher‐magnification HAADF‐STEM images of the SrTiO_3_/SiO_x_ interface viewed along the SrTiO_3_ [010] and [1$\bar{1}$0] zone axes, respectively, with corresponding atomic‐resolution insets highlighting the transition from crystalline SrTiO_3_ to the amorphous SiO_x_ layer.

Figure 2b,c shows zoomed‐in cross‐sectional HAADF images from two different regions of the sample taken along the [010] and [1$\bar{1}$0] zone axes of SrTiO_3_, respectively, revealing sharp, well‐defined interfaces between KNbO_3_ and SrTiO_3_ in both regions. The resolved atomic‐column arrangements supports a local epitaxial orientation relationship between KNbO_3_ and SrTiO_3_. In contrast, the interface between SrTiO_3_ and SiO*_x_* exhibits a distinctly different structure (Figure 2d,e). An amorphous layer is observed in the immediate vicinity of the SiO*_x_* interface consistent with previous reports of strontium silicide formation [27] at the interface between SrTiO_3_ and SiO_*x*_ as observed from EDX measurements (Figures S5 and S6). Additionally, SrTiO_3_ can amorphize when grown on SiO_*x*_ above 600°C [30, 31]. This amorphous layer, however, is confined only to the near‐interface region, with the SrTiO_3_ transitioning to a fully crystalline structure as the distance from the SiO_x_ interface increases. This transition from amorphous to crystalline SrTiO_3_ demonstrates that despite the interfacial disorder caused by silicon oxidation and the presence of SiO*_x_*, the SrTiO_3_ buffer layer successfully establishes high‐quality crystalline growth that serves as an effective template for subsequent KNbO_3_ epitaxy. The onset of the amorphous SrTiO_3_ layer at the SiO_x_ interface has previously been observed to occur at growth temperatures above 600°C, with the layer exceeding 100 nm in thickness for growth temperatures above 800°C, validating the necessity of a thick SrTiO_3_ buffer layer [30].

Additionally, the TEM images reveal local atomic steps at the KNbO_3_/SrTiO_3_ interface (Figure 2c) and localized variations in atomic‐column contrast near the interface and within the SrTiO_3_ buffer layer (Figure S7). Because the imaged region represents a projection through the specimen thickness (∼50−100nm), atomic columns from different depths along the beam direction can overlap in projection, producing KNbO_3_ – SrTiO_3_ overlap patterns and apparent interfacial local distortion in the image. These distortions are hypothesized to arise from a combination of interfacial strain, which produces relative lattice offsets across the interface (Figures S8 and S9), and localized defects, such as threading dislocations and out‐of‐phase boundaries, in the SrTiO_3_ layer, similar to those previously reported for SrTiO_3_ buffer layers grown on silicon [17]. These interfacial structural features and their possible origins are further elaborated in the Supporting Information.

### 1.3 Phase Transitions in KNbO_3_

To experimentally identify the phase transitions in KNbO_3_ grown on SrTiO_3_/Si temperature‐dependent second‐harmonic generation (SHG) measurements were performed. Electric dipole SHG is a nonlinear optical process, where incident light at frequency ω, is converted into second‐harmonic light at twice the frequency, 2ω, reflected off the sample. Only materials with a broken inversion symmetry show an SHG response. Figure 3a shows a schematic of the SHG setup used for experiments. Additionally, by rotating the polarization of the incident light, polar plots can be generated by measuring the two orthogonal polarizations (*p* and *s* polarized) of the second‐harmonic light. Modelling the SHG polar plots reveals the point group symmetry of the material.

**FIGURE 3:**
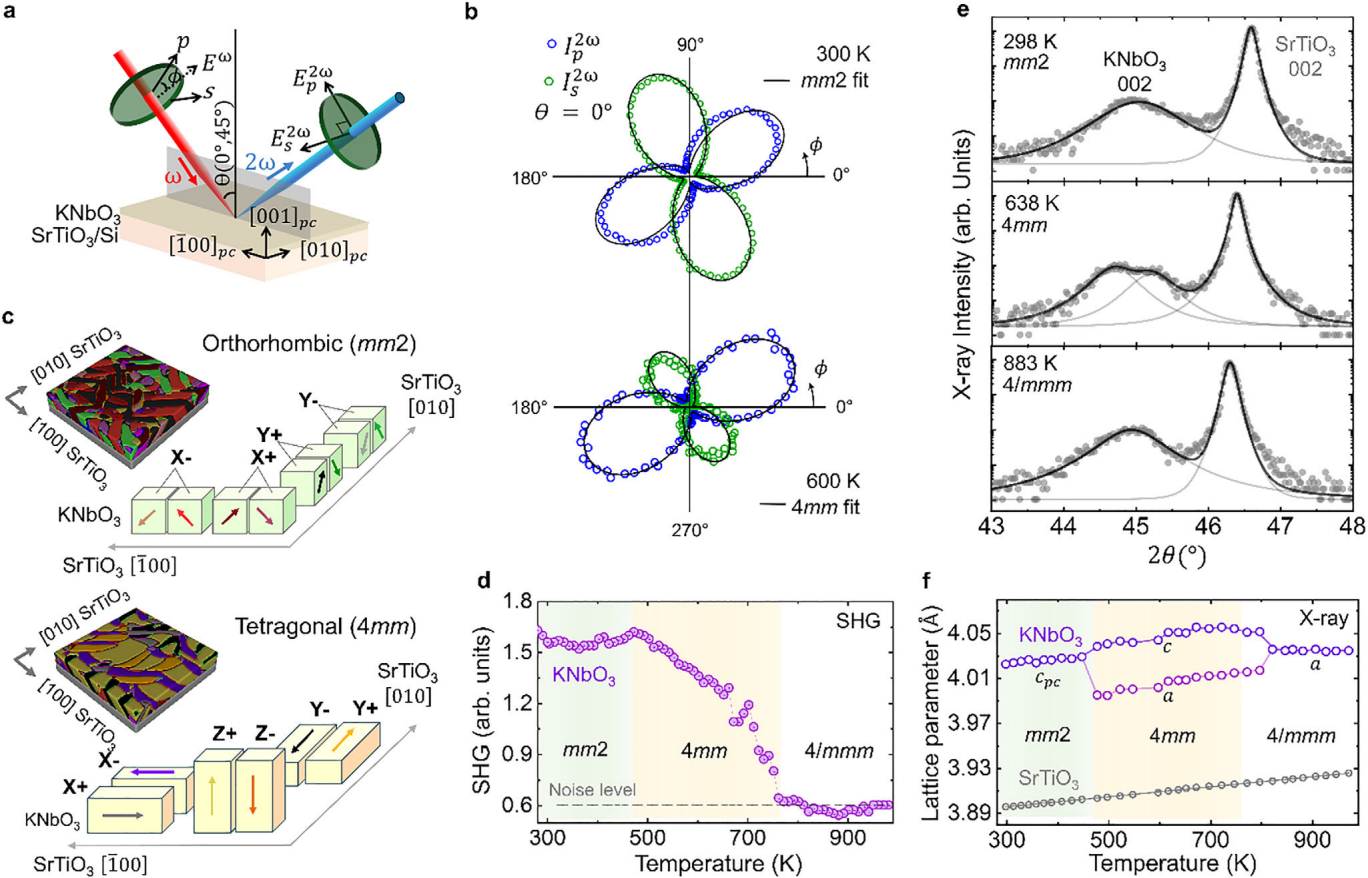
(a) Schematic of second‐harmonic generation (SHG) setup for both normal (θ = 0°) and oblique (θ = 45°) incidence SHG measurements. (b) Normal incidence (θ = 0°) polar plots of KNbO_3_ on SrTiO_3_/Si measured at 300 and 600 K. 300 K polar plot is fitted to a multidomain orthorhombic (point group *mm*2) model, while a multidomain tetragonal (point group 4*mm*) model is used to fit polar plots at 600 K. Fitting equations are derived in Supporting Information. (c) Multidomain orthorhombic domain structure used to fit SHG polar plots at 300 K. Inset shows the phase‐field microstructure at 450 K, the temperature where the orthorhombic phase fraction is maximized (∼80%) in predictions (upper panel). While in general eight orthorhombic domain variants are possible, as discussed later through PFM measurements, the films have a built‐in electric field which prefers a unique out‐of‐plane polarization component. This results in only four domains: *X* +,  *X* −,  *Y* +,  *Y* −, each with only one out‐of‐plane polarization component. Multidomain tetragonal domain structure used to fit SHG polar plots at 600 K. Inset shows phase‐field predicted microstructure at 600 K where the predicted phase fraction of the tetragonal phase is dominant with a phase fraction of ∼75%. (lower panel) (d) Temperature‐dependent SHG intensity ($I_{p}^{2\omega }\left(\phi =0^{\circ }\right)$) measured at normal incidence for KNbO_3_ on SrTiO_3_/Si. (e) Lab θ − 2θ XRD scans around the 002 SrTiO_3_ peak. A Pearson VII function is used to fit the XRD peaks [32] (see Supporting Information). At 298 K, the 002 KNbO_3_ peak is characterized by a single peak, while evolves into a double peak at 638 K (tetragonal phase), eventually becoming a single peak at 883 K (cubic phase). (f) Extracted temperature‐dependent lattice parameter of KNbO_3_ and SrTiO_3_.

Figure 3b shows normal incidence SHG polar plots of KNbO_3_ grown on SrTiO_3_/Si at 300 and 600 K. The room temperature SHG polarimetry is fitted to a multidomain orthorhombic (point group *mm*2) model, the expected room temperature phase for single crystal KNbO_3_. As confirmed from x‐ray measurements, the KNbO_3_ film are strain‐relaxed and epitaxial with the SrTiO_3_ buffer layer; in pseudocubic (*pc*) geometry KNbO_3_ [100]_pc_ || [100] SrTiO_3_. In this geometry, the orthorhombic (*or*) unit cell of KNbO_3_ can be defined with axes parallel to the [110]_pc_, ${\left[1\bar{1}0\right]}_{\mathrm{pc}},$ and [001]_pc_ pseudocubic directions [33]. The ferroelectric polarization, parallel to the [010]_or_ (|| [101]_pc_) direction, is 45° with respect to the film surface giving a total of 8 possible domain variants [34, 35] as shown in Figure 3c. As discussed later through PFM measurements, however, a built‐in electric field exists in the films which prefers a unique out‐of‐plane polarization direction, reducing the total domain variants to 4. Since the polarization has both in and out‐of‐plane components, both oblique and normal incidence SHG response is measurable (Figure S10). The four possible domain variants can be crystallographically represented as: KNbO_3_ [100]_pc_, ${\left[\bar{1}00\right]}_{\mathrm{pc}}$, [010]_pc_ or ${\left[0\bar{1}0\right]}_{\mathrm{pc}}$ || [100] SrTiO_3_ depending on their in‐plane polarization directions and can be labelled as *X* +, *X* −, *Y* + and *Y* − with area fractions *A*_X +_,  *A*_X −_,  *A*_Y +_  and *A*_Y −_, respectively, where *A*_X +_ + *A*_X −_ + *A*_Y +_ +  *A*_Y −_ = 1. The out‐of‐plane polarization component of each of these domains is pointed along the same direction, (either all along [001]_pc_ or ${\left[00\bar{1}\right]}_{\mathrm{pc}}$) due to built‐in electric field (discussed later). In normal incidence, the SHG polar plots can be described by the following general equation [36] (see Supporting Information):

(1) $$\begin{array}{ccc} I_{i}^{2\omega } & = & K_{1i}{\left(\mathrm{co}s^{2}\theta +K_{2i}\mathrm{si}n^{2}\theta \right)}^{2}+K_{3i}\mathrm{si}n^{2}2\theta \\ & + & K_{4i}\left(\mathrm{co}s^{2}\theta +K_{2i}\mathrm{si}n^{2}\theta \right)sin2\theta \end{array}$$

where *i* = *p* or *s* depending on the measured SHG polarization. The nonlinear optical coefficients, *d*_ij_ (in Voigt notation) of KNbO_3_ can be related to the extracted fit parameters as follows:

(2) $$\;\frac{d_{0}}{d_{32}}=K_{2s}=\frac{1}{K_{2p}}\;$$

$$\;{\left(\frac{d_{24}}{d_{0}}\right)}^{4}=\frac{K_{3s}K_{3p}}{K_{1s}K_{1p}K_{2s}^{2}}$$

$$\;{\left(\frac{\delta A_{X}}{\delta A_{Y}}\right)}^{4}=\frac{K_{3s}K_{1p}}{K_{1s}K_{3p}K_{2s}^{2}}$$

$$\mathrm{co}s^{2}\Gamma =\frac{K_{4p}^{2}}{4K_{1p}K_{3p}}=\frac{K_{4s}^{2}}{4K_{1s}K_{3s}}$$

where, *d*_0_ = *d*_15_  + (*d*_33_ + *d*_31_)/2, δ *A*_X(Y)_ = *A*_X(Y) +_   − *A*_X(Y) −_ and $\Gamma =\left(4\pi l/\lambda \right)\left[n_{3}^{O,2\omega }-n_{0}^{O,2\omega }\right]$ is the phase shift in the second‐harmonic light generated between domains *X* + /*X* −  and *Y* + /*Y* − due to the birefringence of the film. Here, $n_{0}^{O,2\omega }=\sqrt{2/[{\left(1/n_{1}^{O,2\omega }\right)}^{2}+{\left(1/n_{2}^{O,2\omega }\right)}^{2}]}$, with $n_{1}^{O}$, $n_{2}^{O}$, $n_{3}^{O}$ representing the principal refractive indices of the orthorhombic unit cell and *l* is the film thickness. The extracted parameters from KNbO_3_ on SrTiO_3_/Si are tabulated in Table 1 in comparison with single crystal KNbO_3_ reference values [36]. The close match between the measured and expected parameters confirms the orthorhombic phase of unstrained KNbO_3_ grown on SrTiO_3_/Si at room temperature. Additionally, δ*A*_X_/δ *A*_Y_ =  0.8 ± 0.1 reflects an almost equal net poling area fraction of KNbO_3_ along both the [100] and [010] directions of the SrTiO_3_ buffer layer. Oblique incidence polar plots can also be similarly fitted with the same general Equation (1), where the K‐parameters involve different convolutions of the nonlinear optical coefficients (Figure S10).

**TABLE 1:** Extracted SHG polar plot fit parameters at 300 K (orthorhombic fit) and 600 K (tetragonal fit). Parameters at 300 K are compared to KNbO_3_ single crystal reference at 300 K [36]. Parameters at 600 K are compared to reference values on KNbO_3_ on SrTiO_3_/MgO films measured at 573 K [35].

	Orthorhombic			Tetragonal	
Orthorhombic parameters 300 K	**KNbO_3_ on SrTiO_3_/Si** **(300 K)**	**Reference (Single crystal, 300 K)** [36]	**Tetragonal** **Parameters** **(600 K)**	**KNbO_3_ on SrTiO_3_/Si** **(600 K)**	**Reference (Thin film 573 K)** [35]
***d***_0_/***d***_32_	2.8 ± 0.3	2.56	***d***_33_/***d***_31_	2.1 ± 0.3	2.5 ± 0.3
***d***_24_/***d***_0_	0.5 ± 0.1	0.45	***d***_15_/***d***_33_	0.4 ± 0.1	0.4 ± 0.1
***δA***_**X**_/***δA***_**Y**_	0.8 ± 0.1	—	***δA***_**X**_/***δA***_**Y**_	1.4 ± 0.1	1.5 ± 0.2
**cos** ^* *^ **Γ**	cos Γ_p_ = 0.8 ± 0.1 cos Γ_s_ = 0.7 ± 0.1	0.86	**cos** ^* *^ **Γ**	cos Γ_*p*_ = 0.5 ± 0.1 cos Γ_*s*_ = 0.8 ± 0.1	0.8 ± 0.2

In contrast, to fit the SHG polarimetry at 600 K a multidomain tetragonal (point group 4*mm*) model was used, the expected phase for single crystal KNbO_3_. As shown in Figure 3c, six different tetragonal domain variants are considered depending on the orientation of the polarization direction of the KNbO_3_ unit cell (along [001]) with respect to the SrTiO_3_ buffer layer. Since *Z* + and *Z* − domains are polarized out‐of‐plane, they do not contribute to a normal incidence SHG signal. From the remaining domains (*X* +,  *X* −,  *Y* +,  *Y* −) with area fractions *A*_X +_,  *A*_X −_,  *A*_Y +_  and *A*_Y −_, respectively, expressions to fit the SHG polar plots can be derived to have the same general form as Equation (1), but with different relationship between the *K*‐parameters and nonlinear optical coefficients as shown below (see Supporting Information);

(3) $$\;\frac{d_{33}}{d_{31}}=K_{2s}=\frac{1}{K_{2p}}\;$$

$$\;{\left(\frac{d_{15}}{d_{33}}\right)}^{4}=\frac{K_{3s}K_{3p}}{K_{1s}K_{1p}K_{2s}^{2}}$$

$$\;{\left(\frac{\delta A_{X}}{\delta A_{Y}}\right)}^{4}=\frac{K_{3s}K_{1p}}{K_{1s}K_{3p}K_{2s}^{2}}$$

$$\mathrm{co}s^{2}\Gamma =\frac{K_{4p}^{2}}{4K_{1p}K_{3p}}=\frac{K_{4s}^{2}}{4K_{1s}K_{3s}}$$

where, δ *A*_X(Y)_ = *A*_X(Y) +_   − *A*_X(Y) −_ and $\Gamma =\left(4\pi l/\lambda \right)\left[n_{3}^{T,2\omega }-n_{1}^{T,2\omega }\right]$ with $n_{1}^{T}$, $n_{2}^{T}\left(=n_{1}^{T}\right)$, *n*_3_ representing the principal refractive indices of the tetragonal unit cell. The extracted parameters are tabulated in Table 1 and compared to previous reports on strain relaxed tetragonal KNbO_3_ thin films at 573 K, showing a close match between them.

It is important to note that while the extracted tensor coefficient ratios agree well with single crystal and thin film references for both the tetragonal and orthorhombic phases, the same general form of Equation (1) can fit both the polar plots. Hence, to conclusively elucidate the phase transition, temperature‐dependent x‐ray diffraction measurements were performed to measure the out‐of‐plane lattice parameter of the KNbO_3_ film. Across the transition, the 002 KNbO_3_ film peak evolves from a single peak in the orthorhombic phase to a double peak in the tetragonal phase eventually becoming a single peak again in the cubic phase (Figure 3e). All orthorhombic unit cells have the same out‐of‐plane lattice parameter, while in the tetragonal phase unit cells with out‐of‐plane (*Z* ±) and in‐plane polarization (*X* ±,  *Y* ±) have different out‐of‐plane lattice parameters. Figure 3f shows the temperature evolution of the out‐of‐plane lattice parameter showing the tetragonal‐to‐orthorhombic phase transition temperature. This has also been observed previously in strain‐relaxed KNbO_3_ thin films on SrTiO_3_ substrate [32].

Figure 3d shows the temperature‐dependent SHG response of KNbO_3_ on SrTiO_3_/Si. The SHG intensity decreases in the tetragonal phase. This is because in the orthorhombic phase, all domain variants generate SHG signal in normal incidence, while in the tetragonal phase only domain variants with in‐plane polarization are SHG active [35, 37]. SHG measurements show an orthorhombic‐to‐tetragonal transition at ∼475 K and tetragonal‐to‐cubic at ∼763 K, in agreement with x‐ray diffraction measurements. In comparison, single crystal KNbO_3_ transitions from orthorhombic to tetragonal at ∼492 K and from tetragonal to cubic at ∼676 K. To further understand the temperature‐dependent phase and microstructure evolution, phase‐field simulations were performed for strain‐relaxed KNbO_3_ thin films on SrTiO_3_/Si. Figure S11shows the predicted phase fraction evolution and transition temperatures of the KNbO_3_ film in agreement with experimental observations. Additionally, rather than exhibiting discrete phase transitions, the simulations reveal mixed phase regions at the transition where one phase gradually grows at the expense of the other. Inset of Figure 3c and Figures S12 and S13 show the temperature evolution of the microstructure of the KNbO_3_ film, and the averaged polarization averaged across multiple domains.

To confirm the multi‐domain nature of the KNbO_3_ films, room temperature piezo‐response force microscopy (PFM) was performed using an interferometric beam displacement sensing PFM (IDS‐PFM). Unlike conventional PFM, which utilizes an optical beam deflection system and measures displacement through an angular measurement of the cantilever, IDS‐PFM uses an interferometric detection system to measure the absolute physical displacement at the tip‐sample contact point [38, 39]. This eliminates global capacitive crosstalk from the cantilever body and enables direct measurement of electromechanical displacement free of electrostatic artifacts (see Supporting Information).

IDS‐PFM measurements confirm a spatially varying piezoelectric response with a maximum response reaching $∼\;\;10\;\mathrm{pm}\;V^{-1}$ across the film (Figure S14). A box‐in‐box poling experiment, however, reveals no domain switching contrast (Figure S14). We attribute this to the absence of a bottom electrode in the KNbO_3_ film grown on ∼100 nm SrTiO_3_ buffer layer on silicon. Additional, further switching spectroscopy PFM (SS‐PFM) measurements show that whilst points do not indicate reliable switching in both directions, a few are suggestive of switching occurring on one side of the hysteresis loop, resulting in finite switchable polarization and work of switching at those sites (Figure S15).

Additionally, IDS‐PFM measurements show a unimodal pixel density distribution centered around 0° phase with no secondary peak at 180° phase difference (Figure S16). A uniform vertical PFM phase across the measured region is consistent with the presence of a built‐in electric field (imprint) in the KNbO_3_ film, which preferentially stabilizes one out‐of‐plane polarization orientation over the other (either along [001]_pc_ or ${\left[00\bar{1}\right]}_{\mathrm{pc}}$). This has previously been observed in BaTiO_3_ films grown on SrTiO_3_/Si as well and is attributed to asymmetric charge screening between the top (air) and bottom (SrTiO_3_/Si) interface or inhomogeneous defect dipoles and surface defects [25, 40]. Phase‐field simulations microstructural simulations does not such include a built‐in field and hence shows domain variants with either out‐of‐plane polarization orientation (Figure 3c).

To further elucidate the domain inhomogeneities, SHG microscopy was performed at room temperature. Based on multidomain orthorhombic model (Equation (1)), by taking SHG microscopy at different combinations of the input and output polarizations, ratios of SHG tensor coefficients and area fraction ratios (δ*A*_X_/δ*A*_Y_) can be extracted (see Supporting Information). Figure 4a shows the spatial variation of the *d*_24_/*d*_32_ SHG tensor coefficients with a mean value of 1.4 in agreement with single crystal KNbO_3_ reference values. Maps of other coefficient ratios are shown in Figures S17–19. The spatial variation of the area fraction ratio δ*A*_X_/δ*A*_Y_ with a mean value of 0.8 is shown in Figure 4b. Region with δ*A*_X_/δ*A*_Y_ < 1, represent a net poling along the [010] direction of the SrTiO_3_ buffer layer while regions with δ*A*_X_/δ*A*_Y_ > 1 represent a net poling along [100] direction. Figure 4c shows the heat map of the joint distribution of *d*_24_/*d*_32_ versus δ*A*_X_/δ*A*_Y_.

**FIGURE 4:**
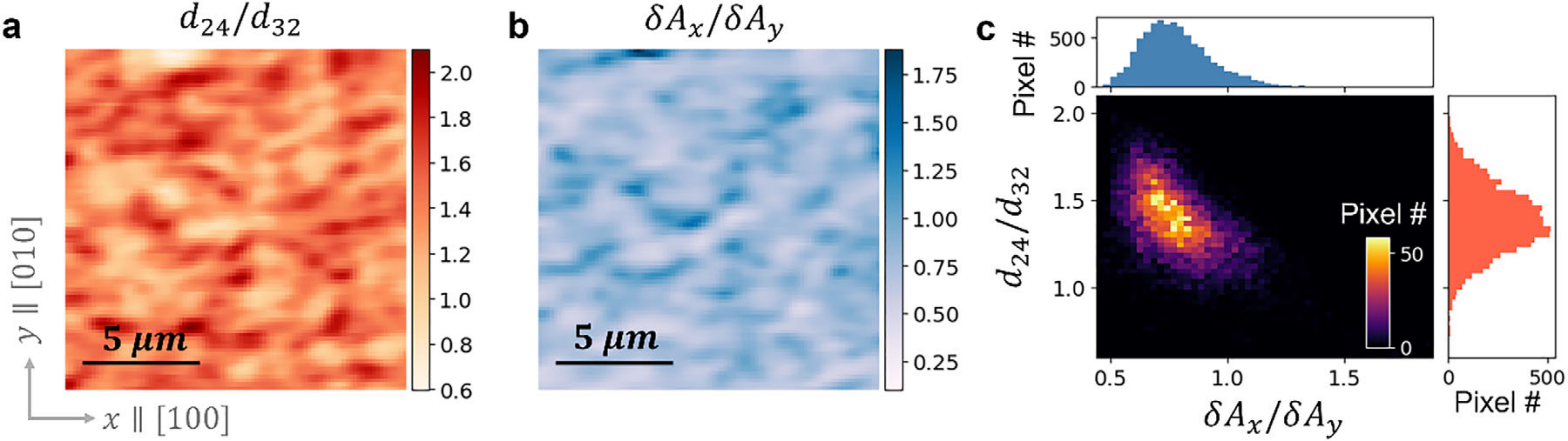
(a) Map of *d*_24_/*d*_32_ SHG tensor coefficient ratio measured from SHG microscopy (see Supporting Information) across a 15 µm × 15 µm region with a mean *d*_24_/ *d*_32_ =  1.4 in agreement with bulk KNbO_3_ reference values. The *x* and *y*‐directions shown correspond to the [100] and [010] direction of the SrTiO_3_ buffer layer. (b) Corresponding map of δ*A*_x_/δ*A*_y_ across the same region with a mean δ*A*_x_/δ*A*_y_ = 0.8, showing regions preferentially poled along [100] or [010]. (c) Pixel correlated *d*_24_/*d*_32_ versus δ*A*_x_/δ*A*_y_ depicted as a heat map of the pixel density. Insets show the pixel count histogram of the individual axes showing unimodal distributions.

### 1.4 Electro‐Optic Response of KNbO_3_ on SrTiO_3_/Si

The electro‐optic effect describes a change in the refractive index, *n*, under an applied electric field *E*, given by $\Delta {\left(\frac{1}{n^{2}}\right)}_{\mathrm{ij}}=r_{\mathrm{ijk}}\;E_{k}$, where *r*_ijk_ is the Pockels tensor describing the linear electro‐optic effect and *i*, *j*, *k* represent dummy subscripts related to the crystal physics axes of the material. Room temperature electro‐optic measurements of KNbO_3_ on SrTiO_3_/Si were performed at 1550 nm using a polarizer‐sample‐compensator‐analyzer (PSCA) setup [41] as shown in Figure 5a. In this technique, changes in the transmitted intensity of light through a pair of cross polarizers is measured as a function of an applied electric field to the sample. 500 µm square electrodes of 5 nm Ti and 150 nm Pt, with 200 µm spacing were lithographically patterned to apply electric field in the in‐plane direction parallel to the [100] direction of the SrTiO_3_ buffer layer. COMSOL simulations verifying the field distribution between the electrodes are shown in Figure S20.

**FIGURE 5:**
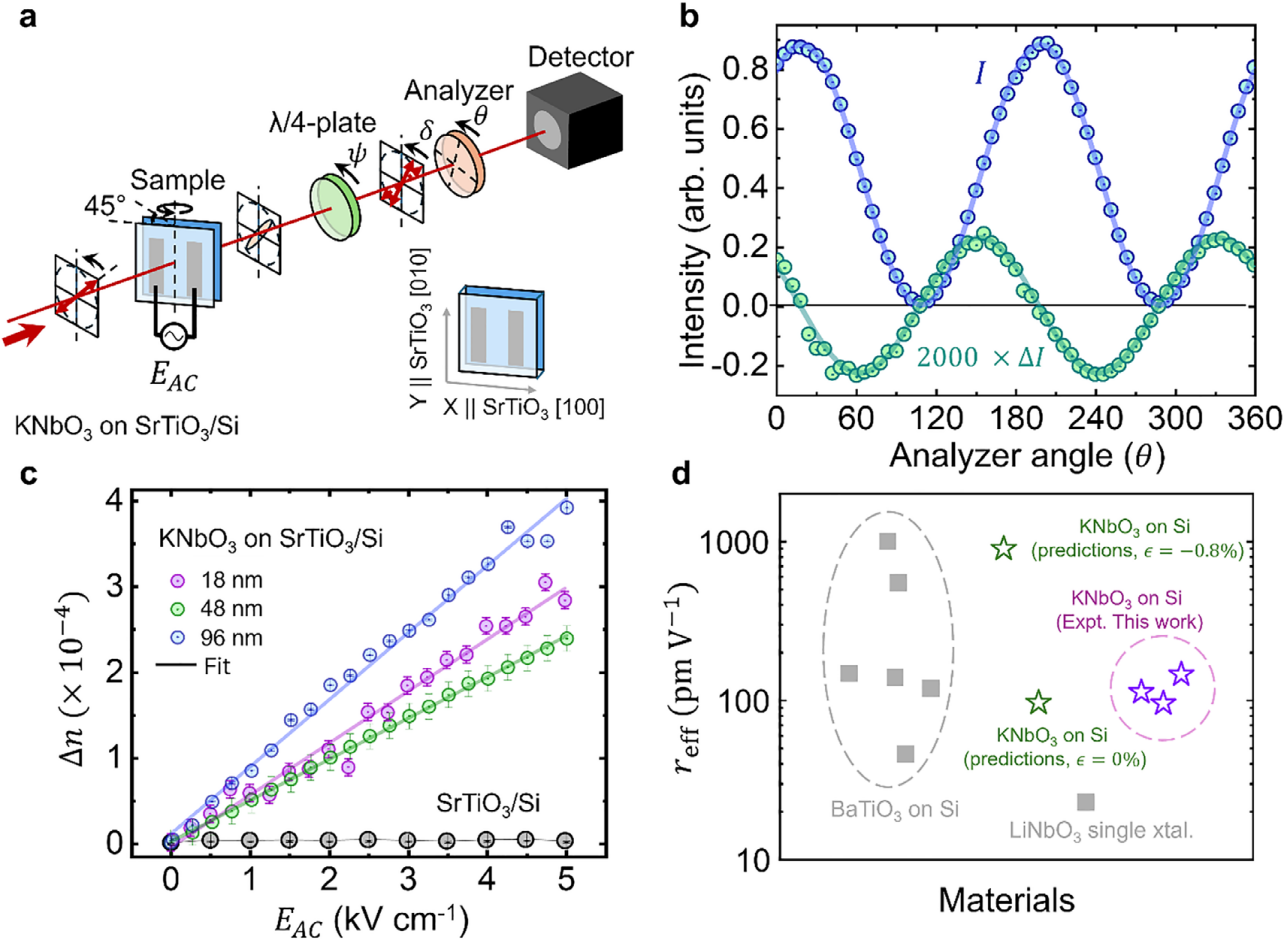
(a) Schematic of the electro‐optics setup used for measurements. Inset shows the crystallographic direction of the SrTiO_3_ buffer layer in laboratory frame (*X*,  *Y*,  *Z*). (b) Unmodulated and modulated transmission intensities through the KNbO_3_ on SrTiO_3_/Si sample. The modulated intensity was measured under a 2 kV cm^−1^ and 555 Hz AC electric field. (c) Change in refractive index (Δ*n*) versus applied AC electric field amplitude (*E*_AC_) measured in three different KNbO_3_ film thicknesses grown on SrTiO_3_/Si and compared to the SrTiO_3_/Si substrate. (d) Comparison of room temperature *r*_eff_ for KNbO_3_ on SrTiO_3_/Si and other related materials measured using the same technique in transmission geometry (Table S2). The phase‐field predicted microstructure averaged electro‐optic tensor is converted to *r*_eff_ based on experimental measurement geometry (see Supporting Information). A modest compressive strain of − 0.8% is predicted to enhance *r*_eff_ to $∼\;\;900\;\mathrm{pm}\;V^{-1}$. This is compared to thin films of BaTiO_3_ on Si [2, 42, 43, 44, 45, 46], and LiNbO_3_ [41] single crystals.

The incident polarization of light is set to lie along the [110]_pc_ (|| [110] SrTiO_3_) of the KNbO_3_ film with the sample rotated 45° away from the incident probe direction. Without an electric field, the unmodulated transmission of light can be described as;

(4) $$I=I_{0}\;\cos^{2}\left(\phi +\delta \right)$$

where, ϕ represents the angle through which the analyzer is rotated and δ is the unmodulated phase accumulated by the beam upon transmitting through the sample and other optical components as shown in Figure 5a. Under an applied electric field, the modulated transmitted intensity, Δ*I*(*E*), can be expressed as;

(5) $$\Delta I\;\left(E\right)=I_{0}\;\Delta \delta \left(E\right)\;\sin2\left(\phi +\delta \right)$$

Figure 5b shows the unmodulated and modulated transmitted intensities at 2 kV cm^−1^ fitted to Equations (4) and (5), respectively. As expected from Equation (4), the modulated intensity maximizes at an analyzer angle corresponding to half transmission of the unmodulated intensity. The induced phase change, Δδ(*E*), can be converted into electric field induced change in birefringence, $\Delta n=\frac{\Delta \delta \lambda }{2\pi L}$, where λ = 1550 nm and *L* = *t*/ *cos* θ_film_ is the path length of light through the sample with *t* representing the film thickness and θ_film_ is the angle of beam propagation inside the KNbO_3_ film (see Supporting Information). An effective electro‐optic coefficient is derived according to $r_{\mathrm{eff}}=\frac{2\Delta n}{n^{3}E}=113\;\pm 12\;\mathrm{pm}\;V^{-1}$, for a 18 nm thick film, where *n* = 2.20 is the refractive index of KNbO_3_ at 1550 nm [5].

To investigate the effect of thickness, electro‐optic measurements on thicker KNbO_3_ thin films (48 and 96 nm) were performed. As shown in Figure 5c, a linear response is observed in all measured thicknesses signifying Pockels effect as the origin of the electro‐optic response and yields an *r*_eff_ =  91 ± 5 pm V^−1^ for the 48 nm film and 146 ± 14 pm V^−1^ for the 96 nm film in comparison to 113 ± 12 pm V^−1^ for the 18 nm film. All three thicknesses possess similar electro‐optic response, which is expected given the strain‐relaxed nature of all the three films measured. Electro‐optic measurements performed on SrTiO_3_/Si show no electric field induced change in Δ*n* confirming the electro‐optic response to originate in the KNbO_3_ film alone.

Additionally, the experimentally measured electro‐optic response closely matches the phase‐field predicted microstructure‐averaged electro‐optic response of KNbO_3_ grown on SrTiO_3_/Si at 300 K converted to a *r*_eff_ =  88 pm V^−1^ (Figure 5d), based on experimental geometry (see Supporting Information). The predicted temperature evolution of the electro‐optic coefficients is shown in Figure S13. Phase‐field simulated wavelength dispersion of the electro‐optic response is shown in Figure S21. While the strain‐relaxed films established here yield *r*_eff_ = 146 ± 14 pm V^−1^, phase‐field simulations reveal substantial room for improvement through epitaxial strain engineering. Under compressive strain, a monoclinic phase is stabilized at low temperatures while a tetragonal phase persists at high temperatures, with the tetragonal‐to‐monoclinic phase transition temperature tunable by an epitaxial strain. At the phase boundary, large enhancements in the electro‐optic response are predicted, reaching up to $r_{\mathrm{eff}}\;∼\;\;900\;\mathrm{pm}\;V^{-1}$ at 300 K for a − 0.8% compressive strain. This enhancement can also be achieved through compositional tuning in the (K,Na)NbO_3_ system, where sodium substitution introduces a chemical strain that drives analogous phase boundary engineering and electro‐optic enhancement [7].

This behavior can be understood within the thermodynamic theory of the electro‐optic effect. For example, the *r*_51_ (*r*_131_ in uncontracted notation) electro‐optic coefficient can be expressed as [41, 47]:

(6) $$\;r_{131}=\left(\frac{\partial B_{13}}{\partial E_{1}}\right)\;≅\left(\frac{\partial B_{13}}{\partial P_{1}^{L}}\right)\;\left(\frac{\partial P_{1}^{L}}{\partial E_{1}}\right)=f_{131}^{L}\;\chi_{11}^{L}$$

where $B_{\mathrm{ij}}={\left(\frac{1}{n^{2}}\right)}_{\mathrm{ij}}$ is the optical dielectric stiffness, $P_{i}^{L}$ is the lattice polarization, $f_{131}^{L}$ is the linear polar‐optic coefficient, and $\chi_{11}^{L}$ is the lattice dielectric susceptibility. Since $f_{131}^{L}$ is proportional to the spontaneous polarization and remains roughly constant under compressive strain, the enhancement is primarily driven by the increase of $\chi_{11}^{L}$ near the phase boundary. By adjusting the compressive strain or composition, the temperature window of this enhancement can be positioned at any desired operating temperature [5, 41].

## 2 Conclusion

Our results show that high‐quality, crystalline KNbO_3_ films can be grown on silicon substrates using SrTiO_3_ buffer layers via suboxide molecular‐beam epitaxy. X‐ray diffraction and transmission electron microscopy confirm epitaxy with excellent crystalline quality, while the films exhibit strain‐relaxed characteristics. Second‐harmonic generation measurements and x‐ray diffraction supported by phase‐field simulations, reveal phase transitions and nonlinear optical coefficients comparable to single crystal KNbO_3_, consistent with the relaxed strain state of the films. Significantly, electro‐optic measurements demonstrate a response of 146 ± 14 pm V^−1^, confirming that KNbO_3_ retains substantial electro‐optic activity when integrated on silicon.

As shown in Figure 5d, phase‐field predictions further suggest that under modest compressive strain, the electro‐optic response can be enhanced to $∼\;\;900\;\mathrm{pm}\;V^{-1}$[8]. This strain tunability can be achieved on silicon platforms by employing compositionally graded (Ba,Sr)TiO_3_ [23, 24] buffer layers instead of pure SrTiO_3_. By varying the barium concentration, the lattice parameter of the buffer layer can be systematically tuned, imposing controlled epitaxial strain on the KNbO_3_ film. Alternatively, compositionally graded SrSn_x_Ti_1‐x_O_3_ or SrGe_x_Ti_1‐x_O_3_ buffer layers can also be used to achieve tunable lattice parameters required to strain the KNbO_3_ film [25, 26].

KNbO_3_’s intrinsically strong piezoelectric response and electrostrictive effect [8], combined with the epitaxial silicon integration demonstrated here, provides a pathway towards on‐chip acoustic wave devices and piezo‐optomechanical systems [15]. The nonlinear optical properties demonstrated through second‐harmonic generation suggest potential applications in frequency conversion and all‐optical signal processing integrated directly with silicon photonic circuits [48], particularly transceivers of interest currently for optical interconnects employed in data centers. Furthermore, the ferroelectric nature of this material system opens possibilities for non‐volatile memory elements integrated with photonic and electronic components, enabling novel computing architectures that merge logic, memory, and optical interconnects on a single chip.

## 3 Methods

### 3.1 Thin Film Synthesis and Characterization

#### 3.1.1 KNbO_3_ on SrTiO_3_ Substrates

KNbO_3_ on SrTiO_3_ films were grown using a Vecco Gen 10 MBE system, with a 10.6 µm CO_2_ laser substrate heater from Epiray GmbH (THERMALAS Substrate heater). A description of the preparation of the Nb_2_O_5_, potassium effusion cells, flux calibration with quartz crystal microbalance (QCM), RHEED pattern monitoring and growth of KNbO_3_ thin films on GdScO_3_ and DyScO_3_ substrates is elaborated elsewhere [8].

#### 3.1.2 Epitaxial Thin Films of SrTiO_3_ on Si

100 nm of SrTiO_3_ was deposited on a 3‐inch (300 µm thick) Si wafer which was prepared according to the detailed description reported previously [17]. The Si wafer was deoxidized with a buffered HF solution before deposition. 2.5 Unit cells of SrTiO_3_ were deposited at 300°C followed by an anneal at 500°C. This process was repeated 3 times before the remaining 249 unit cells were deposited at 550°C at a background pressure of 8.5 × 10^−9^ torr resulting in a total film thickness of 100 nm. After deposition, the wafer was diced into 10 × 10 mm^2^ pieces, cleaned with acetone, IPA and deionized water before loading individual 10 × 10 mm^2^ pieces back into the MBE system for subsequent deposition of KNbO_3_.

### 3.2 Thermodynamic Simulation

Thermodynamic calculations were carried out using the Thermo‐Calc software [49] and the SGTE Substance Database (SSUB5) [50]. Missing thermodynamic data for KNbO_3_, KNb_3_O_8_, K_4_Nb_6_O_17_, and KNb_8_O_14_ were incorporated into SSUB5 using density‐functional‐theory (DFT) based data from the Materials Project [51]. These values were implemented through the following formation reactions:

(R1) $$K_{2}O+Nb_{2}O_{5}=2\;\mathrm{KNb}O_{3}$$

(R2) $$K_{2}O+3\;Nb_{2}O_{5}=2\;\mathrm{KN}b_{3}O_{8}$$

(R3) $$2K_{2}O+3\;Nb_{2}O_{5}=K_{4}Nb_{6}O_{17}$$

(R4) $$K_{2}O+6\;\mathrm{NbO}+8\;\mathrm{Nb}O_{2}+Nb_{2}O_{5}=2\;\mathrm{KN}b_{8}O_{14}$$

The reaction Gibbs energies obtained from the Materials Project [50] for Reactions (R1)–(R4) are −34.5, −17.2, −27.9, and −10.7 kJ/mol‐atom, respectively.

Figure 1a shows the calculated pressure‐temperature phase diagram for the K‐Nb‐O system at a fixed O_2_ partial pressure of 7.5 ×10^−4^ Torr (corresponding to an oxygen activity of 10^−6^). Under these conditions, the stability window for KNbO_3_ lies near the center of the diagram. As an example, Figure S1 shows the calculated K‐Nb‐O2 isothermal phase diagram at 1000 K under a total pressure of 1 atm calculated using the present thermodynamic database.

### 3.3 Synchrotron Based X‐Ray Reciprocal Space Mapping

Synchrotron x‐ray diffraction measurements were performed at the ID4B (QM2) beamline of Cornell high energy synchrotron source (CHESS) at an incident x‐ray energy was 20 keV with an area detector array (Pilatus 6 M). This is further elaborated elsewhere [8, 41].

### 3.4 Transmission Electron Microscopy

The sample lamellae were fabricated using a Thermo Fisher Scientific Scios 2 dual‐beam FIB–SEM. To protect the surface during milling, two Pt capping layers were deposited: electron‐beam deposition (3.2 nA) followed by Ga ion‐beam deposition (0.26 nA). Two lamellae, each ∼1 µm thick, were lifted out from the region of interest with the in‐plane x‐direction aligned to the SrTiO_3_ [100] and [110] directions, respectively, and mounted on a half‐grid. To reduce ion‐beam damage, the lamella was thinned under Ga ions while progressively lowering the accelerating voltage from 30 to 2 kV (30, 16, 8, 5, and 2 kV). Both specimens reached an estimated thickness of ∼70 nm in the target area.

Cross‐sectional STEM measurements were carried out using an aberration‐corrected 300 kV FEI Titan G2 60/300 S/TEM. The microscope was operated with a 70 µm C2 condenser aperture, spot size 8, 115 mm camera length, and a probe convergence semi‐angle of 25.2 mrad. HAADF signals were recorded over 42–244 mrad, and images were obtained with a probe current of 0.07 nA. EDS data were acquired using a Super‐X detector array, and the spectra were processed in Bruker Esprit software. Post‐processing of the EDS maps included 1/4 Q‐map binning, spectral deconvolution via series‐fit, and quantitative analysis using P/B ZAF method. For visualization, brightness and gamma setting were applied to every elemental channel to maintain consistent rendering.

### 3.5 Second‐Harmonic Generation Measurements

Temperature‐dependent second‐harmonic generation (SHG) polarimetry were measured using at a fundamental wavelength of 800 nm generated by a Spectra‐Physics Solstice Ace Ti:Sapphire laser with an 80 MHz repetition rate and 300 fs pulse width. Room temperature SHG microscopy was performed in normal incidence reflection geometry with the fundamental laser beam focused by a high‐NA objective (0.75 NA). This is further elaborated elsewhere [8, 41].

### 3.6 Phase‐Field Simulations

Phase‐field simulations of the structural and optical properties are discussed in detail elsewhere [5, 41]. A table containing all coefficients used in the phase‐field simulations relevant for KNbO_3_ is given in the supplementary material (Table S1). For the simulations, a system size of 128 ∆x_1_ × 128 ∆x_2_ × 36 ∆x_3_ is used. There are 12 ∆x_3_ grid points for the substrate where the elastic constants are assumed to be the same as the ferroelectric film, and there are 9 ∆x_3_ grid points acting as an air layer above the film. The thickness of KNbO_3_ is set to be 15 ∆x_3_. Periodic boundary conditions are used along the in‐plane lateral directions, and natural boundary conditions are used along the film‐substrate and film‐air interfaces. The interface between the film and the substrate is assumed to be relaxed, and hence the in‐plane misfit strain is set to:

$$\varepsilon_{11}=0,\varepsilon_{22}=0,\varepsilon_{12}=\varepsilon_{21}=0$$

The simulations begin with an initial condition of P_i_^L^ (x_i_, t = 0) = [0.0 0.0 0.1 C m^−2^] + ∆P^noise^ (x_i_,t = 0), where, P^i^_L_ (x_i_,t) is the lattice polarization and |P^noise^| = 0.1 C m^−2^, and relax for 20,000 time steps. O simulate the evolution of the polarization under an applied electric field and the corresponding electro‐optic effect, a uniform electric field is applied along the [100]_pc_ direction ranging from −70 kV cm^−1^ to 70 kV cm^−1^ which varies in time with a sinewave, with one period of the being equal to 65,000 time steps. The linear electro‐optic effect is found by taking the average of the refractive index over the ferroelectric material as a function of an applied electric field and fitting to a cubic polynomial centred at zero. The phase fractions were determined by sorting the local lattice polarization vectors according to the minimum angular deviation from the ideal crystallographic polarization directions assuming the presence of only tetragonal, orthorhombic and rhombohedral domains. The lattice–polarization evolution was computed using the Mu‐Pro phase‐field simulation package.

### 3.7 Electro‐Optic Measurement

Electro‐optic measurements were performed using a homebuilt polarizer‐sample‐compensator‐analyzer (PSCA) setup with a 1550 nm HP 81689A continuous‐wave laser source. A Nirvana 2017 balanced detector was used for measurements with Stanford SR830 lock‐in amplifier. An AC modulation was applied to the sample at 555 Hz frequency with an Agilent 33220A signal generator amplified by a TREK 610C high voltage amplifier. This is further elaborated elsewhere [41].

## Author Contributions

**Sankalpa Hazra**: conceptualization, investigation, writing – original draft, writing – review and editing, visualization, methodology, validation, data curation, formal analysis. **Tobias Schwaigert**: conceptualization, investigation, visualization, writing – original draft, writing – review and editing, validation, methodology. **Yingxin Zhu**: investigation, visualization, formal analysis. **Aiden Ross**: investigation, visualization, formal analysis, writing – review and editing, software. **Muhammad Aminul Haque Chowdhury**: investigation. **Shun‐Li Shang**: investigation, writing – review and editing, formal analysis. **Sameer Singh**: investigation. **Zhiyu Zhang**: investigation. **Zhe Wang**: investigation. **Brynn Brower**: investigation. **Saugata Sarker**: investigation. **Suchismita Sarker**: investigation, funding acquisition, resources, writing – review and editing. **Zi‐Kui Liu**: resources, supervision, writing – review and editing, funding acquisition. **Long‐Qing Chen**: resources, supervision, funding acquisition, formal analysis, writing – review and editing. **Yang Yang**: investigation, writing – review and editing, supervision, funding acquisition. **Darrell G. Schlom**: conceptualization, writing – original draft, validation, writing – review and editing, supervision, project administration. **Venkatraman Gopalan**: conceptualization, investigation, funding acquisition, writing – review and editing, project administration, supervision, resources.

## Conflicts of Interest

The authors have a provisional patent filed on this work. Long‐Qing Chen has a financial interest in MuPRO, LLC, a company which licenses and markets the software package used in this research.

## Supporting information




**Supporting File**: adma75067‐sup‐0001‐SuppMat.docx.

## Data Availability

The data that supports the findings of this study are available within the article. Datasets used for the generation of figures presented in the article is available at https://doi.org/10.6084/m9.figshare.33920134. Additional data related to the film growth and structural characterization is available at https://doi.org/10.34863/ssec‐tz95.
