# Supplementary material for: Epitaxially Integrated Electro‐Optic KNbO3 Thin Films on Silicon
Source: Adv Mater. 2026 Sep 28;38(63):e75067. doi: 10.1002/adma.75067 (PMC13755664; doi:10.1002/adma.75067)
Supplement: Supplementary file 1 [file ADMA-38-e75067-s001.docx]

**Supporting Information**

**Epitaxially integrated Electro-Optic KNbO_3_ thin films on Silicon**

Sankalpa Hazra*, Tobias Schwaigert*, Yingxin Zhu, Aiden Ross, Muhammad Aminul Haque Chowdhury, Shun-Li Shang, Sameer Singh, Zhiyu Zhang, Zhe Wang, Brynn Brower, Saugata Sarker, Suchismita Sarker, Zi-Kui Liu, Rama K Vasudevan, Long-Qing Chen, Yang Yang, Darrell G. Schlom and Venkatraman Gopalan.

**Figure S1: a)** Calculated isothermal phase diagram of the K-Nb-O_2_ system at 1000 K and under a total pressure of 1 atm. Solid phases are denoted with “S”, liquid phases with “L”, and all ternary oxides are all solids. **b)** Thermodynamically calculated potassium overpressure as a function of growth temperature showing predicted adsorption-controlled growth window for KNbO_3_ (shaded region), bounded by potassium-rich and potassium-deficient phase conditions. Theoretically calculated potassium partial pressures required for KNbO_3_ growth (right axis, **Figure 1a, Main Text**) are calculated by adding the predicted potassium overpressure to the potassium partial pressure corresponding to the potassium flux that is being consumed to form KNbO_3_ (which is equal to the niobium flux used for KNbO_3_ growth).

**Note 1: Potassium partial pressure during KNbO_3_ growth**

We derive the relation between the molecular beam flux $F_{K}$ in atoms cm^–2^s^–1^ of potassium and the corresponding equivalent pressure $P_{K}$ based on kinetic gas theory, following the treatment commonly used for molecular-beam epitaxy^[1–3]^.

For a dilute gas, the beam density $N_{K}$ in number of atoms cm^–3^ is related to the pressure $P_{K}$ by the ideal gas law,

$$\begin{matrix} & N_{i}=\frac{P_{i}}{k_{B}T_{i}}, & & \text{(1)} \end{matrix}$$

where $k_{B}$ is the Boltzmann constant and $T_{K}$ is the temperature of the potassium sources. The mean speed of potassium $\bar{c}_{K}$ is given by the Maxwell–Boltzmann distribution,

$$\begin{matrix} & \bar{c}_{i}=\sqrt{\frac{8k_{B}T_{i}}{\pi m_{i}}}, & & \text{(2)} \end{matrix}$$

where $m_{K}$ the molecular mass of potassium. In an ideal gas with isotropic velocity distribution, the potassium flux incident on a surface is $N_{K}\bar{c}_{K}/4$. In contrast, a molecular beam produced by an effusion cell consists of particles traveling predominantly in a single direction. For such a directed beam, the flux $F_{K}$ incident on a surface whose normal is tilted by an angle $\theta$ relative to the beam axis is

$$\begin{matrix} & F_{i}=N_{i}\bar{c}_{i}\cos\theta. & & \text{(3)} \end{matrix}$$

Here, $\theta$ is the angle between the beam propagation direction and the surface normal. For a Vecco Gen 10 MBE this angle is 40°. Substituting Eq. (1) and (2) into (3) and solving for $P_{K}$ gives:

$$\begin{matrix} & P_{i}=\frac{F_{i}}{\cos\theta}\sqrt{\frac{\pi m_{i}k_{B}T_{i}}{8}} & & \text{(4)} \end{matrix}$$

where

- $P_{K}$ is the equivalent pressure of species $i$,
- $F_{K}$is the molecular beam flux (atoms cm^–2^×s^–1^),
- $m_{K}$ is the particle mass,
- $T_{K}$ is the beam temperature,
- $\theta$ is the beam incidence angle relative to the surface normal, and
- $k_{B}$is the Boltzmann constant.

**Figure S2: a)** RHEED pattern of KNbO_3_ grown on SrTiO_3_/Si along the [10] azimuth direction taken post growth at the growth temperature (675 °C). **b)** X-ray rocking curve measured around SrTiO_3_ 001 peak for the SrTiO_3_/Si substrate. **c)** KNbO_3_ 001 rocking curve fit with two components (modeled by Pearson VII functions, see **Note 7**) to separate the sharp central feature from the broad diffuse base, yielding FWHM = 0.0097° for the sharp component and FWHM = 0.7497° for the broad component.

**Figure S3:** Large area reciprocal space maps (RSM) of KNbO_3_ on SrTiO_3_/Si along **a)** $Q_{y}$ and **b)** $Q_{x}$ reciprocal space directions showing epitaxial relationship between KNbO_3_ and SrTiO_3_ buffer layer.

**Figure S4:** AFM maps taken over a $10 \mu m\times10 \mu m$ area of the KNbO_3_ films showing RMS surface roughness of **a)** 1.47 nm for 18 nm thick KNbO3 film and **b)** 6.83 nm for a 96 nm thick KNbO_3_ film. **c)** AFM maps over a $5 \mu m\times5 \mu m$ area of the SrTiO3 film on Si showing RMS surface roughness of 0.97 nm.

**Figure S5:** Cross-sectional scanning transmission electron microscopy (STEM) high-angle annular dark-field (HAADF) image of the KNbO_3_/SrTiO_3_/Si stack taken at a different region of the sample. The accompanying energy-dispersive X-ray spectroscopy (EDS) elemental maps (K, Nb, Sr, Ti, O, Si, and Pt) are acquired from the same field of view and illustrate the elemental distributions across the heterostructure. The KNbO_3_/SrTiO_3_ interface **(a)**, SrTiO_3_/SiO_x_ interface **(b)** and KNbO_3_/SrTiO_3_/Si stack **(c)** are shown.

**Figure S6:** Cross-sectional scanning transmission electron microscopy (STEM) high-angle annular dark-field (HAADF) image of the KNbO_3_/SrTiO_3_/Si stack taken at a different region of the sample. The accompanying energy-dispersive X-ray spectroscopy (EDS) elemental maps (K, Nb, Sr, Ti, O, Si, and Pt) are acquired from the same field of view and illustrate the elemental distributions across the heterostructure. The KNbO_3_/SrTiO_3_ interface **(a)**, SrTiO_3_/SiO_x_ interface **(b)** and KNbO_3_/SrTiO_3_/Si stack **(c)** are shown.

**Note 2: Defect analysis from TEM images**

Several defect regions at the interface between KNbO_3_ and SrTiO_3_ and the SrTiO_3_ buffer layer itself is highlighted in **Figure S7a**. The interface shown in this image contains local atomic steps and structurally distorted regions. There may be several possible origins of these local structural features.

**Figure S7: a)** HAADF-STEM images of the KNbO_3_/SrTiO_3_ interface viewed along the SrTiO_3_ [1$\overline{1}$0] zone axis. A noticeable feature is the existence of atomic step edges at the interface, indicating that the interface is not perfectly flat. **b)** Zoomed in KNbO_3_/SrTiO_3_ interface showing two features: the KNbO_3_/SrTiO_3_ overlapped regions, and the elliptical atomic column images (green box). **c)** Schematic drawing showing the origin of the overlapped regions in (b), due to the roughness and steps at the interface and sample thickness. **d)** Enlarged illustration of the boxed region in (b), highlighting the distorted/elliptical atomic column images of Sr, suggesting that the SrTiO_3_ may contain local defects that lead to misorientation of the crystal with respect to the electron beam direction.

First, there is a “overlapped region” at the interface, as shown in **Figure S7b**. The TEM specimen is not atomically thin; it was prepared by focused ion beam milling and has an estimated thickness of approximately 50–100 nm. Therefore, the image represents a projection of structural information through the specimen thickness (**Figure S7c**). In this case, the observed atomic steps likely indicate that the interface is not perfectly flat through the thickness direction, which is expected for a real interface. As a result, regions near the interface may contain projected contributions from both the top and bottom layers along the electron beam direction, leading to apparent local distortions or overlapping regions in the image (**Figure S7 b-c**).

Second, the atomic structure at the interface contains local structural distortions. For example, the arrow in **Figure S7b** highlights the shift of a SrTiO_3_ cell. For another example, **Figure S7d** shows distorted/elliptical Sr atomic column images. These structural distortions could be resulted from the following reasons.

- Lattice mismatch and strain accommodation across the interface: To examine this possibility, we performed geometric phase analysis (GPA) of the image (**Figure S8**). The GPA strain maps show that the top and bottom layers exhibit different strain states in both the in-plane and out-of-plane directions, indicating that the interface mismatch produces relative lattice offsets across the interface. When the two layers are projected together through the sample thickness, even a small relative displacement between the lattices can produce locally shifted or distorted atomic columns, which may contribute to the features observed near the interface in **Figure S7**.

**Figure S8:** **a)** HAADF-STEM image of the KNbO_3_/SrTiO_3_ heterostructure after auto-cropping to a square field of view. **b)** Vertical strain map ($\varepsilon_{yy}$) computed by GPA using the KNbO_3_ lattice as reference, showing tensile strain (red) in the KNbO_3_ region and compressive strain (blue) in the SrTiO_3_ region, with a locally enhanced strain at the interface exceeding that of either bulk layer. **c)** Vertical strain map ($\varepsilon_{yy}$) using the SrTiO_3_ lattice as reference, confirming the lattice mismatch between the two materials. **d)** Horizontal strain map ($\varepsilon_{xx}$) using the KNbO_3_ lattice as reference. Note that GPA reveals variations in the projected lattice periodicity relative to the selected reference region. Across the heterointerface, these variations may include contributions from lattice mismatch, local deformation, and changes in atomic-column contrast. The interfacial signal is therefore not assigned uniquely to elastic strain.

- Existence of planar defects: we performed fast Fourier transform (FFT) analysis of the top layer, bottom layer, and interfacial region (**Figure S9**). The FFT patterns from the top and bottom regions show similar crystalline structures. In contrast, the FFT pattern from the interfacial region contains streaking features, suggesting the possible presence of planar defects, such as stacking faults, near the interface^[4]^. Such defects are reasonable in this material system and may further contribute to the local structural distortion. Because the selected region is narrow and irregularly bounded, finite-window and masking effects may contribute substantially to this feature. The present FFT analysis does not uniquely identify planar defects.
- Presence of interfacial dislocations or other local defects: A previous study of similarly grown SrTiO_3_/Si films reported threading dislocations and out-of-phase boundaries ^[5]^. Such defects are plausible contributors to the contrast variations observed here, but their identities and densities have not been quantified in the present specimens. Defects such as interfacial dislocations, as well as possible point defects, may also contribute to the distorted contrast observed in the current cross-sectional image.

**Figure S9: a-c)** HAADF-STEM images of the KNbO_3_ region, interface region, and SrTiO_3_ region, respectively. Regions outside each selected area were masked prior to the FFT analysis. **d)** FFT pattern of the KNbO_3_ region, showing a well-defined periodic spot pattern characteristic of the KNbO_3_ lattice. **e)** FFT pattern of the interface region. **f)** FFT pattern of the SrTiO_3_ region, showing well-defined periodic diffraction spots consistent with the SrTiO_3_ lattice.

**Note 3: Derivation of SHG fitting equations for room temperature orthorhombic model**

**Figure 3a, Main text** shows a schematic of the SHG setup used for our experiments. For our modeling, we define a lab coordinate system $(X, Y, Z)$ that is attached to the direction of the incoming beam, with X || *p* polarization, Y || *s* polarization (**Figure 3a, Main text**). In addition, we define a lab coordinate system ($L_{1}=\left[ 100 \right]_{L}$, $L_{2}=\left[ 010 \right]_{L}$, $L_{3}=\left[ 001 \right]_{L}$) attached to the SrTiO_3_ buffer layer where $L_{1} || [100]$, $L_{2} || [010]$ and $L_{3} || [001]$ direction of the SrTiO_3_ substrate.

In the lab coordinate system $(X, Y, Z)$, the electric field of the incident fundamental beam can be expressed as $(E_{o}\cos\phi, E_{o}sin \phi, 0)$ where $\phi$ is the angle through which the polarization is rotated by a half wave plate as shown in **Figure 3a**. For an incidence angle $\theta$ on the sample (see **Figure 3a**), the electric field of the incident beam can be transformed to the lab coordinate system and expressed as $\left( E_{o}\cos\phi\cos\theta,E_{o}\sin\phi,-E_{o}\cos\phi\sin\theta) \right.$.

The incident electric field induces a nonlinear polarization, *P^2ω^*, which can be expressed as:

$P_{i}^{2\omega}\propto d_{ijk}E_{j}^{\omega}E_{k}^{\omega}$	(5)

where, *d_ijk_* is the nonlinear susceptibility tensor and is related to the symmetry of the material under observation and $i,j,k$ are dummy subscripts related to the crystal physics direction of the material. The proportionality constant depends on incident beam fluence, Fresnel’s coefficients at the film-air and film-substrate interfaces and the thickness of the films.

In crystal physics coordinates of the orthorhombic unit cell, *i =* (1, 2, 3), the nonlinear susceptibility tensor of a *mm*2 unit cell can be written in Voigt notation as:

$d_{ij}=\left( \begin{matrix} 0 & 0 & 0 & 0 & d_{15} & 0 \\ 0 & 0 & 0 & d_{24} & 0 & 0 \\ d_{31} & d_{32} & d_{33} & 0 & 0 & 0 \end{matrix} \right)$	(6)

We assume 8 domain variants for modelling the SHG polarimetry as shown in **Figure 3c**. For each of the domains, the relation between the crystal physics direction *i =* (1, 2, 3) and the lab coordinates are specified below

Domain 1: $\left[ \bar{1}01 \right]_{L}$ || 1, $\left[ 0\bar{1}0 \right]_{L}$ || 2, $\left[ 101 \right]_{L}$ || 3

Domain 2: $\left[ \bar{1}0\bar{1} \right]_{L}$ || 1, $\left[ 010 \right]_{L}$ || 2, $\left[ 10\bar{1} \right]_{L}$|| 3

Domain 3:$\left[ 101 \right]$*_L_* || 1, $\left[ 010 \right]$*_L_* || 2, $\left[ \bar{1}01 \right]$*_L_* || 3

Domain 4:$\left[ 10\bar{1} \right]$*_L_* || 1, $\left[ 0\bar{1}0 \right]$*_L_* || 2, $\left[ \bar{1}0\bar{1} \right]$*_L_* || 3

Domain 5: $\left[ 0\bar{1}1 \right]$*_L_* || 1, $\left[ 100 \right]$*_L_* || 2, $\left[ 011 \right]$*_L_* *|*| 3

Domain 6: $\left[ 0\bar{1}\bar{1} \right]$*_L_* || 1, $\left[ \bar{1}00 \right]$*_L_* || 2, $\left[ 01\bar{1} \right]$*_L_* *|*| 3

Domain 7: $\left[ 011 \right]$*_L_* || 1, $\left[ 100 \right]$*_L_* || 2, $\left[ 0\bar{1}1 \right]$*_L_* *|*| 3

Domain 8: $\left[ 0\bar{1}1 \right]$*_L_* || 1, $\left[ \bar{1}00 \right]$*_L_* || 2, $\left[ 0\bar{1}\bar{1} \right]$*_L_* *|*| 3

As discussed in the main text, the 8 domain variants can be grouped into 4 pairs depending on the in-plane component of the polarization as follows:

$X+$ : Domain 1, Domain 2 (Area fraction: $A_{X+}$)

$X-$ : Domain 3, Domain 4 (Area fraction: $A_{X-}$)

$Y+$ : Domain 5, Domain 6 (Area fraction: $A_{Y+}$)

$Y-$ : Domain 7, Domain 8 (Area fraction: $A_{Y-}$)

Normal incidence ($\theta=0^{\circ}$)

Using **Equation 5**, the induced nonlinear polarization can be calculated in the lab coordinate system for each domain pair as expressed below:

|  | $P_{p}^{2\omega}$ | $P_{s}^{2\omega}$ |
| --- | --- | --- |
| Domain X+ | $d^{0}\cos^{2}\phi+d_{32}\sin^{2}\phi$ | $d_{24}\sin2\phi$ |
| Domain X- | $-(d^{0}\cos^{2}\phi+d_{32}\sin^{2}\phi)$ | ${-d}_{24}\sin2\phi$ |
| Domain Y+ | $d_{24}\sin2\phi$ | $d^{0}\sin^{2}\phi+d_{32}\cos^{2}\phi$ |
| Domain Y- | $-d_{24}\sin2\phi$ | $-(d^{0}\sin^{2}\phi+d_{32}\cos^{2}\phi)$ |

The net SHG intensity can be expressed as follows:

$$P_{p}^{2\omega}\propto\left( A^{X+}P_{p}^{2\omega,X+}+A^{X-}P_{p}^{2\omega, X-}+\left( A^{Y+}P_{p}^{2\omega,Y+}+A^{Y-}P_{p}^{2\omega,Y-} \right)e^{i\Gamma} \right)$$

where, $\Gamma=(4\pi l/\lambda)\left[ n_{3}^{O,2\omega}-n_{0}^{O,2\omega} \right]$ is the phase shift generated due to the birefringence of the film. Here, $n_{0}^{O,2\omega}=\sqrt{2/\left[ \left( 1/n_{1}^{O,2\omega} \right)^{2}+\left( 1/n_{2}^{O,2\omega} \right)^{2} \right]}$, with $n_{1}^{O}$, $n_{2}^{O}$, $n_{3}^{O}$ representing the principle refractive indices of the orthorhombic unit cell and $l$ is the film thickness. The generated SHG intensity can be calculated as

$$I_{p}^{2\omega}=P_{p}^{2\omega}{(P}_{p}^{2\omega}){}^{*}=\begin{aligned} \delta A_{X}^{2}\left( P_{p}^{2\omega,X+} \right)^{2}+\delta A_{Y}^{2}\left( P_{p}^{2\omega,Y+} \right)^{2}+2\delta A_{x}\delta A_{y}P_{p}^{2\omega,X+}P_{p}^{2\omega,Y+}\cos\Gamma, \end{aligned}$$

This can be simplified to:

$I_{p}^{2\omega}\propto K_{1p}\left( {cos}^{2}\phi+K_{2p}{sin}^{2}\phi\right)^{2}+K_{3p}{sin}^{2}2\phi+K_{4p}\left( {cos}^{2}\phi+K_{2p}{sin}^{2}\phi\right)sin2\phi$	(7)

where,

$$K_{1p}=\delta A_{X}^{2}d_{0}^{2} K_{2p}=\frac{d_{32}}{d_{0}} K_{3p}=\delta A_{Y}^{2}d_{24}^{2} K_{4p}=2\delta A_{X}\delta A_{Y}d_{0}d_{24}\cos\Gamma$$

$d^{0}=d_{15}+\frac{d_{33}+d_{31}}{2},\delta A_{X} = A_{X+} - A_{X-}$and $\delta A_{Y} = A_{Y+} - A_{Y-}$. Similar expressions can also be derived for $I_{s}^{2\omega}$ where the equivalent coefficients can be expressed as:

$$K_{1s}=\delta A_{Y}^{2}d_{32}^{2} K_{2s}=\frac{d_{0}}{d_{32}} K_{3s}=\delta A_{X}^{2}d_{24}^{2} K_{4s}=2\delta A_{X}\delta A_{Y}d_{32}d_{24}\cos\Gamma$$

Similar expressions have also been shown earlier for multidomain *mm*2 model in epitaxial thin films. Normal incidence SHG polar plots of KNbO_3_ on SrTiO_3_/Si fitted to a multidomain orthorhombic model is shown in **Figure 3b, Main Text**.

Oblique incidence ($\theta=45^{\circ}$):

Like the normal incidence case, the oblique incidence SHG can be modeled in the same formulation and can be simplified to a similar form as **Equation 7**. The coefficients for this case can be expressed as follows:

$$\begin{aligned} &K_{1p}=\left[ \left( A_{X+}+A_{Y+} \right)\left( 2d_{31}+2d_{33}-4d_{15} \right)+A_{X-}d_{32} \right]^{2} \end{aligned}$$

$$\begin{aligned} K_{2p}=\frac{1}{\sqrt{K_{1p}}}\left[ \frac{A_{X+}+A_{Y+}}{8}\left( 2d_{15}-4d_{24}+d_{31}+2d_{32}+d_{33} \right)+A_{X-}d_{31} \right] \end{aligned}$$

$$K_{3p}=2\left( A_{X+}-A_{Y+} \right)^{2}\left( 2d_{24}+d_{31}-d_{33} \right)^{2}$$

$$K_{4p}=2\sqrt{K_{1p}K_{3p}} cos \Gamma$$

$$K_{1s}=\frac{\left( A_{X+}-A_{Y+} \right)^{2}}{4}\left( 2d_{15}+d_{31}+d_{33} \right)^{2}$$

$$K_{2s}=\frac{1}{4\sqrt{2K_{1s}}}\left( A_{X+}-A_{Y+} \right)\left( 2d_{32}-2d_{15}+d_{31}+d_{33} \right)$$

$$K_{3s}=\frac{1}{8}\left[ \left( A_{X+}+A_{Y+} \right)\left( d_{31}-d_{33} \right)-2\sqrt{2}A_{Y-}d_{24} \right]^{2}$$

$$K_{4s}=2\sqrt{K_{1s}K_{3s}}\cos\Gamma$$

Oblique incidence SHG polar plots fitted to a multidomain orthorhombic model is shown in **Figure S10, Supporting Information**.

**Note 4: Derivation of SHG fitting equations for high temperature tetragonal model**

Following a similar prescription, in crystal physics coordinates *i =* (1, 2, 3), the nonlinear susceptibility tensor of a 4*mm* unit cell can be written as:

$d_{ij}=\left( \begin{matrix} 0 & 0 & 0 & 0 & d_{15} & 0 \\ 0 & 0 & 0 & d_{15} & 0 & 0 \\ d_{31} & d_{31} & d_{33} & 0 & 0 & 0 \end{matrix} \right)$	(8)

In this case we assume 6 domain variants for modelling the SHG polarimetry as shown in **Figure 3c**. For each of the domains, the relation between the crystal physics direction *i =* (1, 2, 3) and the lab coordinates are specified below

Domain 1: $\left[ 010 \right]$*_L_* || 1, $\left[ 001 \right]$*_L_* || 2, $\left[ 100 \right]$*_L_* || 3

Domain 2: $\left[ 0\bar{1}0 \right]$*_L_* || 1, $\left[ 00\bar{1} \right]$*_L_* || 2, $\left[ \bar{1}00 \right]$*_L_* || 3

Domain 3:$\left[ 101 \right]$*_L_* || 1, $\left[ 010 \right]$*_L_* || 2, $\left[ 010 \right]$_L_ || 3

Domain 4:$\left[ 10\bar{1} \right]$_L_ || 1, $\left[ 0\bar{1}0 \right]$_L_ || 2, $\left[ 0\bar{1}0 \right]$*_L_* || 3

Domain 5: $\left[ 100 \right]$_L_ || 1, $\left[ 010 \right]$_L_ || 2, $\left[ 001 \right]$*_L_* *|*| 3

Domain 6: $\left[ 0\bar{1}\bar{1} \right]$*_L_* || 1, $\left[ \bar{1}00 \right]$*_L_* || 2, $\left[ 00\bar{1} \right]$*_L_* *|*| 3

Unit cells with polarization direction lying in the out-of-plane direction do not contribute to an SHG response. Hence only 4 domains contribute to an SHG response as described below:

$X+$ : Domain 1(Area fraction: $A_{X+}$)

$X-$ : Domain 2 (Area fraction: $A_{X-}$)

$Y+$ : Domain 3 (Area fraction: $A_{Y+}$)

$Y-$ : Domain 4 (Area fraction: $A_{Y-}$)

Normal incidence ($\theta=0^{\circ}$)

Using Equation 1, the induced nonlinear polarization can be calculated in the lab coordinate system for each domain pair as expressed below:

|  | $P_{p}^{2\omega}$ | $P_{s}^{2\omega}$ |
| --- | --- | --- |
| Domain X+ | $d_{33}\cos^{2}\phi+d_{31}\sin^{2}\phi$ | $d_{15}\sin2\phi$ |
| Domain X- | $-(d_{33}\cos^{2}\phi+d_{31}\sin^{2}\phi)$ | ${-d}_{15}\sin2\phi$ |
| Domain Y+ | $d_{15}\sin2\phi$ | $d_{31}\cos^{2}\phi+d_{33}\sin^{2}\phi$ |
| Domain Y- | $-d_{15}\sin2\phi$ | $-(d_{31}\cos^{2}\phi+d_{33}\sin^{2}\phi)$ |

The net SHG intensity can be expressed as follows:

$$P_{p}^{2\omega}\propto\left( A^{X+}P_{p}^{2\omega,X+}+A^{X-}P_{p}^{2\omega, X-}+\left( A^{Y+}P_{p}^{2\omega,Y+}+A^{Y-}P_{p}^{2\omega,Y-} \right)e^{i\Gamma} \right)$$

where, $\Gamma=(4\pi l/\lambda)\left[ n_{3}^{T,2\omega}-n_{1}^{T,2\omega} \right]$ is the phase shift generated due to the birefringence of the film, with $n_{1}^{T}$, $n_{2}^{T} (= n_{1}^{T})$, $n_{3}^{T}$ representing the principle refractive indices of the tetragonal unit cell and $l$ is the film thickness. The generated SHG intensity has the same general form as Equation 3 with the following relationship between the *K*-parameters and the nonlinear optical coefficients

$$K_{1p}=\delta A_{X}^{2}d_{33}^{2} K_{2p}=\frac{d_{31}}{d_{33}} K_{3p}=\delta A_{Y}^{2}d_{15}^{2} K_{4p}=2\delta A_{X}\delta A_{Y}d_{33}d_{15}\cos\Gamma$$

$$K_{1s}=\delta A_{Y}^{2}d_{31}^{2} K_{2s}=\frac{d_{33}}{d_{31}} K_{3s}=\delta A_{X}^{2}d_{15}^{2} K_{4s}=2\delta A_{X}\delta A_{Y}d_{31}d_{15}\cos\Gamma$$

where, $\delta A_{X} = A_{X+} - A_{X-}$and $\delta A_{Y} = A_{Y+} - A_{Y-}$. Normal incidence SHG plots of KNbO_3_ on SrTiO_3_/Si at 600 K fitted to a multidomain tetragonal model is shown in **Figure 3b, Main Text**.

Oblique incidence ($\theta=45^{\circ}$)

Following a similar prescription fitting equations for oblique incidence polar plots can be generated. For the oblique incidence case, tetragonal unit cells with polarization in the out of plane direction also need to be considered. The derived SHG fitting equations can be expressed as the following:

$$I_{p}^{2\omega}=\left( \frac{\delta A_{X}-\delta A_{Z}}{8} \right)^{2}\left[ \left( d_{33}+d_{31}-2d_{15} \right)\cos^{2}\phi+2d_{31}\sin^{2}\phi\right]^{2}$$

$$\begin{aligned} I_{p}^{2\omega}=\delta A_{X}^{2}d_{31}^{2}&\left( \cos^{2}\phi+\frac{d_{33}}{d_{31}}\sin^{2}\phi\right)^{2}+\frac{\delta A_{z}^{2}d_{15}^{2}}{8}\sin^{2}2\phi+\frac{\delta A_{Y}\delta A_{z}d_{31}d_{15}}{\sqrt{2}}\left( \cos^{2}\phi+\frac{d_{33}}{d_{31}}\sin^{2}\phi\right) \end{aligned}$$

where, $\delta A_{Z}=A_{Z+}-A_{Z-}$. Oblique incidence SHG polar plots of KNbO_3_ on SrTiO_3_/Si at 600 K fitted to a multidomain tetragonal model is shown in **Figure S10, Supporting Information**.

**Figure S10:** Oblique incidence ($\theta=45^{\circ}$) polarimetry of KNbO_3_ on SrTiO_3_/Si at 300 K (fitted to a multidomain orthorhombic model) and 600 K (fitted to a multidomain tetragonal model).

| $g_{1111}^{LL}$ | $27.8\times{10}^{-2}\left( m^{4}/C^{2} \right)$ | $p_{1111}$ | $0.5328$ (Unitless) |
| --- | --- | --- | --- |
| $g_{1122}^{LL}$ | $-1.1\times{10}^{-2}\left( m^{4}/C^{2} \right)$ | $p_{1122}$ | $0.1584$ (Unitless) |
| $g_{1212}^{LL}$ | $8.2\times{10}^{-2}\left( m^{4}/C^{2} \right)$ | $p_{1212}$ | $-0.432$ (Unitless) |
| $a_{11}$ | $a_{o}\left( T_{s}\coth\left( \frac{T_{s}}{T} \right)-T_{s}\coth\left( \frac{T_{s}}{T_{c}} \right) \right)$ | $\mu_{e}$ | $32.8 \times{10}^{-23}\left( \frac{Kg}{m}\frac{m^{4}}{C^{2}} \right)$ |
| $T_{c}$ | $650K$ | $\gamma_{e}$ | $3\times{10}^{-9}\left( \frac{Kg}{ms}\frac{m^{4}}{C^{2}} \right)$ |
| $T_{s}$ | $54K$ | $B_{ij}^{e, ref}\left( T_{0} \right)$ | $0.2546$ (Unitless) |
| $a_{0}$ | $3.53\times{10}^{5}\left( \frac{J}{m^{3}} \frac{m^{4}}{{K C}^{2}} \right)$ | $T_{0}$ | $398K$ |
| $a_{1111}$ | $-5.86\times{10}^{8}\left( \frac{J}{m^{3}} \frac{m^{8}}{C^{4}} \right)$ | $\alpha_{11}$ | $2.657\times{10}^{-5}\left( \frac{1}{K} \right)$ |
| $a_{1122}$ | $9.66\times{10}^{8}\left( \frac{J}{m^{3}} \frac{m^{8}}{C^{4}} \right)$ | $a_{11111111}$ | $1.74\times{10}^{10}\left( \frac{J}{m^{3}} \frac{m^{16}}{C^{8}} \right)$ |
| $a_{111111}$ | $2.71\times{10}^{9}\left( \frac{J}{m^{3}} \frac{m^{12}}{C^{6}} \right)$ | $a_{11111122}$ | $5.99\times{10}^{9}\left( \frac{J}{m^{3}} \frac{m^{16}}{C^{8}} \right)$ |
| $a_{111122}$ | $-2.2\times{10}^{9}$ $\left( \frac{J}{m^{3}} \frac{m^{12}}{C^{6}} \right)$ | $a_{11112222}$ | $2.5\times{10}^{10}\left( \frac{J}{m^{3}} \frac{m^{16}}{C^{8}} \right)$ |
| $a_{112233}$ | $4.4\times{10}^{9}$ $\left( \frac{J}{m^{3}} \frac{m^{12}}{C^{6}} \right)$ | $a_{11112233}$ | $-1.63\times{10}^{10}\left( \frac{J}{m^{3}} \frac{m^{16}}{C^{8}} \right)$ |
| $Q_{1111}$ | $0.11$ $\left( \frac{m^{4}}{C^{2}} \right)$ | $Q_{1122}$ | $-0.053$ $\left( \frac{m^{4}}{C^{2}} \right)$ |
| $Q_{1212}$ | $0.026$ $\left( \frac{m^{4}}{C^{2}} \right)$ |  |  |

**Table S1.** Coefficients in the thermodynamic free energy function and equation of motion used for phase-field simulations^[6]^

**Figure S11:** Phase-field simulated temperature evolution of phase fraction in KNbO_3_ grown on SrTiO_3_/Si.

**Figure S12: a)** Phase field simulations of representative domain microstructures of strain relaxed KNbO_3_ thin films on SrTiO_3_/Si at various temperatures. **b)** The color code of each polarization direction corresponding to each domain variant as a legend to panel a.

**Figure S13: a)** Phase-field predicted temperature evolution of polarization in strain relaxed KNbO_3_ on SrTiO_3_/Si. **b)** Phase-field predicted temperature dependence of microstructure averaged electro-optic coefficients ($r_{\mathrm{xyz}}^{\mathrm{eff}}$) of KNbO_3_ on SrTiO_3_/Si. Here, dummy subscripts ($x, y, z$) refer to laboratory coordinates ($X, Y, Z$) as shown in **Inset, Figure 5a (Main text)**.

**Note 5: PFM switching and spectroscopy**

To understand the origin of the electromechanical response, additional interferometric displacement sensing PFM (IDS-PFM) were performed. Standard optical beam deflection PFM (OBD-PFM) tracks cantilever tilt. This introduces non-local electrostatic crosstalk from the broad cantilever body. In contrast, IDS-PFM measures absolute physical displacement up down displacement of the tip directly over the tip-sample contact point. This effectively eliminates global capacitive crosstalk, while minor local electrostatic interactions can still exist at the tip-sample contact point.

**Figure S14: a)** Topography of a $2 \mu m\times2 \mu m$ region on the KNbO_3_ film across which interferometric displacement sensing PFM (IDS-PFM) were performed. **b)** Vector PFM response ($=Acos\left( \phi\right),$ where A is the measured PFM amplitude and $\phi$ is measured phase) measured before poling. **c)** Written box-in-box voltage pattern applied for poling. **d)** Vector PFM response measured after poling.

IDS-PFM experiments were performed on a $2 \mu m\times2 \mu m$ region on the KNbO_3_ films as shown in **Figure S14a**. As shown in **Figure S14b**, a piezoelectric response can be observed across this region. The non-uniformity of the piezo-response could be related to the surface roughness of the KNbO_3_ film. To test ferroelectric switching, a box-in-box poling experiment was performed by applying a voltage pattern as shown in **Figure S14c**. As shown in **Figure S14d**, the vector PFM response ($=Acos\left( \phi\right),$ where A is the measured PFM amplitude and $\phi$ is measured phase) after poling reveals no reversal contrast across the written voltage pattern, indicating no polarization reversal was observed. We attribute this to the absence of a bottom electrode in the KNbO_3_ film grown on ~ 100 nm SrTiO_3_ buffer layer on silicon.

To further characterize the local electromechanical response, switching spectroscopy PFM (SS-PFM) was performed across the film, with local hysteresis loops fitted using a nine-parameter phenomenological model^[7]^. From this fitting, spatially resolved maps of the switchable polarization and work of switching can be derived as shown in **Figure S15a**. Representative hysteresis loops from two different locations on the film are shown in **Figures S15b** and **S15c**. While some pixels show partial hysteretic behavior with finite switchable polarization and non-zero work of switching, no pixel yielded a clean 180° switching loop was observed across the grid. While no ferroelectric switching could be established, due to the absence of a bottom electrode, the finite switchable polarization and work of switching observed in some regions nonetheless suggest a local electromechanical response beyond pure electrostatic artifact.

**Figure S15: a)** AFM topography and corresponding maps of the switchable polarization and work of switching. Switching spectroscopy-PFM (SS-PFM) is measured across ~5,000 points and is fitted to a nine-parameter phenomenological model. From this fitting, the switchable polarization and work of switching is derived. **b,c)** Representative PFM switching loop measured in two different positions of the sample fitted to the nine-parameter model.

To understand the PFM phase distribution, we analyze the phase as a function of signal-to-noise ratio (SNR) of the piezoelectric amplitude across the measured region (**Figure S16**). The SNR bins separate pixels by the reliability of their piezoelectric response between pixels where the signal is barely above the noise floor (SNR 0–2) and pixels with a high-confidence piezoelectric response (SNR > 10). Importantly, irrespective of the SNR bin, the phase shows a unimodal distribution with a single peak at 0° and no secondary peak at 180°. This confirms the spatially uniform phase previously observed in vertical PFM measurements.

This is consistent with the presence of a built-in electric field (imprint) in the KNbO_3_ film, which preferentially stabilizes one polarization orientation over the other. Such built-in fields have similarly been observed in BaTiO_3_ films grown on SrTiO_3_/Si and can be attributed to asymmetric charge screening between the top (air) and bottom (SrTiO_3_/Si) interfaces, or to inhomogeneous defect dipoles and surface defects. In this case, one polarization direction is favored over the other. For example, the X+ and Y+ variants could be favored over X− and Y−. The phase-field simulations, which do not include such a built-in field, show both domain orientations.

**Figure S16:** Signal-to-noise ratio (SNR)-dependent pixel density distribution of the PFM phase measured for the KNbO_3_ film. The pixels density is binned by the SNR of the piezoelectric amplitude response (SNR 0–2: 18% of pixels; SNR 2–5: 43%; SNR 5–10: 27%; SNR > 10: 13%).

**Note 6: SHG microscopy**

The second-harmonic generation (SHG) intensity measured at polarization channel $i (i = s or p)$ as a function of input polarization angle $\theta$ is given by:

$$I_{i}^{2\omega}=K_{1i}\left( K_{2i}\sin^{2}\theta+\cos^{2}\theta\right)^{2}+K_{3i}\sin^{2}(2\theta)+K_{4i}\left( K_{2i}\sin^{2} \theta+\cos^{2} \theta\right)\sin(2\theta)$$

where $i=p$or$s$ depending on the measured SHG polarization. SHG intensity maps were acquired at four input polarization angles ($\theta= 0^{\circ}, 45^{\circ}, 90^{\circ}, 135^{\circ}$) for both $p$ and $s$ output channels, yielding eight $90\times90$ pixel spatial maps over a $15\times15 \mu m^{2}$ area.

From the above equation, at φ = 0° and φ = 90°,

$$I_{i}\left( 0^{\circ} \right)=K_{1i}$$

$$I_{i}\left( {90}^{\circ} \right)=K_{1i}\cdot K_{2i}^{2}$$

Giving,

$$K_{1i}=I_{i}\left( 0^{\circ} \right)$$

$$K_{2i}=\sqrt{\frac{I_{i}\left( {90}^{\circ} \right)}{I_{i}\left( 0^{\circ} \right)}}$$

Similarly, from $\theta= 45^{\circ}$ and $\theta= 135^{\circ}$;

$$I_{i}\left( {45}^{\circ} \right)+I_{i}\left( {135}^{\circ} \right)=2\left[ K_{1i}\left( \frac{K_{2i}+1}{2} \right)^{2}+K_{3i} \right]$$

$$I_{i}\left( {45}^{\circ} \right)-I_{i}\left( {135}^{\circ} \right)=2K_{4i}\left( \frac{K_{2i}+1}{2} \right)$$

giving,

$$K_{3i}=\frac{I_{i}\left( {45}^{\circ} \right)+I_{i}\left( {135}^{\circ} \right)}{2}-I_{i}\left( 0^{\circ} \right)\left( \frac{\sqrt{I_{i}\left( {90}^{\circ} \right)/I_{i}\left( 0^{\circ} \right)}+1}{2} \right)^{2}$$

$$K_{4i}=\frac{I_{i}\left( {45}^{\circ} \right)-I_{i}\left( {135}^{\circ} \right)}{\sqrt{I_{i}\left( {90}^{\circ} \right)/I_{i}\left( 0^{\circ} \right)}+1}$$

All eight $K$ parameters (corresponding to $p$ or $s$ polarized SHG measured) are extracted pixel-by-pixel from the eight measured intensity maps.

From this, maps of SHG tensor coefficient ratios can be extracted as follows;

$$\begin{aligned} &\frac{d_{0}}{d_{32}}=K_{2s}=\frac{1}{K_{2p}} \\ &\frac{d_{24}}{d_{0}}=\left( \frac{K_{3s}K_{3p}}{K_{1s}K_{1p}K_{2s}^{2}} \right)^{1/4} \\ &\frac{\delta A_{X}}{\delta A_{Y}}=\left( \frac{K_{3s}K_{1p}}{K_{1s}K_{3p}K_{2s}^{2}} \right)^{1/4} \end{aligned}$$

**Figure S17: a)** $d_{0}/d_{32}$computed using ${1/K}_{2p}$. **b)** $d_{0}/d_{32}$computed using $K_{2s}$. **c)** A pixel count histogram of their difference.

Each of these three ratios are computed pixel-by-pixel across the $90\times90$ pixel map, yielding spatially resolved images of $d_{0}/d_{32}, d_{24}/d_{0}, \delta A_{X}/\delta A_{Y}$. Since $d_{0}/d_{32}$ can be estimated independently from both channels ($K_{2s}$ from the $s$-channel and ${1/K}_{2p}$ from the $p$-channel), both estimates were computed and averaged together:

$$\left( \frac{d_{0}}{d_{32}} \right)_{avg}=\frac{1}{2}(K_{2s}+\frac{1}{K_{2p}})$$

**Figure S17 a -** **b** shows $d_{0}/d_{32}$computed using the $K_{2s}$ from the $s$-channel and ${1/K}_{2p}$ from the $p$-channel. A pixel count histogram of their difference is shown in **Figure S17c**, showing the mean difference to be 0 with a mean of 0.02 as expected confirming the validity of the approach. **Figure S18** shows a spatial map of the $d_{24}/d_{0}$ ratio. The combined tensor ratio$\frac{d_{24}}{d_{32}}=\left( \frac{d_{0}}{d_{32}} \right)_{avg}\times\frac{d_{24}}{d_{0}}$ is shown in **Figure 4a,b, Main Text**.

**Figure S19** shows the correlation between $d_{24}/d_{32}$vs. $\delta A_{X}/\delta A_{Y}$. From all 8100 pixels a Perason correlation coefficient of −0.57, indicating a weak spatial anticorrelation between the effective nonlinear tensor ratio and the domain population across the film.

**Figure S18**: Spatial map of the $d_{24}/d_{0}$ ratio.

**Figure S19**: Correlation between $d_{24}/d_{32}$vs. $\delta A_{X}/\delta A_{Y}$.

**Note 7: Temperature-dependent XRD peak fitting**

Temperature-dependent X-ray diffraction scans were collected around the KNbO_3_ 002 film reflection and the adjacent SrTiO_3_ (002) substrate reflection from room temperature to 700 °C. Each scan was fit using Pearson VII functions of the form

$$I(2\theta) = A \left[ 1 + \left( \frac{2\theta- 2\theta_{0}}{w} \right)^{2} \right]^{-m}$$

where $A$, $2\theta_{0}$, $w$, and $m$ represent the amplitude, peak center, width, and shape parameters, respectively, following a similar approach used previously for epitaxial KNbO_3_ thin films grown on SrTiO_3_ substrate^[8]^. At each temperature, the SrTiO_3_ substrate peak and the KNbO₃ film peak(s) were fit simultaneously using a nonlinear least-squares routine, with a linear constant background term included in the model. At temperatures where the KNbO_3_ peak resolved into two components, two Pearson VII profiles were used to capture the film contribution, sharing common width and shape parameters to avoid overfitting. Peak positions were converted to out-of-plane lattice parameters ($d$) using Bragg's law, $d = \lambda/sin(\theta)$ (for the 002 peak), with $\lambda= 1.540598 Å$ (Cu $K_{\alpha}$).

**Figure S20:** Electric field magnitude and electric potential between surface deposited electrodes as simulated in the COMSOL Multiphysics Electrostatics module for two cases. Vector field streamlines have been added to the electric potential visualization as a guide to the eye. The simulation environment consists of an 18 nm KNbO_3_ layer on a 200 nm SrTiO_3_ layer on top of a 200 µm Si layer. Their dielectric constants are 884^[9]^, 200^[10]^ and 11.7^[11]^ respectively. Two 400 μm square surfaces are defined on the film surface, with one (right) set to ground and the other (left). The simulations are performed for two cases: **a)** Applied voltage of 20 V and **b)** applied voltage of 100 V. The COMSOL calculated electric field value in the electrode gap for both cases matches the expected value obtained when dividing the voltage applied by the electrode separation. The dotted ellipses indicate the relative full width half max diameter (~ 44.8 $\mu m$ calculated using knife-edge measurements) of the focused beam used for electro-optic characterization, projected onto the film surface at 45° incidence.

**Figure S21:** Phase-field simulated wavelength ($\lambda$) dispersion of electro-optic tensor coefficients, **a)** $r_{11}$, **b)** $r_{31}$, **c)** $r_{51}$. **d)** The simulated tensor coefficients are converted to $r_{\mathrm{eff}}$ based on experimental geometries used for measurements (see **Note 8**).

## **Note 8: Refractive index and effective electro-optic coefficient (**$\boldsymbol{r}_{\mathbf{eff}}\boldsymbol{)}$ **from phase-field**

**Refractive index**

The phase-field simulations yield the full electro-optic tensor coefficients $r_{11}, r_{31},$ and $r_{51}$ (in Voigt notation). In the experimental geometry, the measured quantity is an effective EO coefficient $r_{\mathrm{eff}}$ that depends on the crystal orientation, the beam propagation direction, and the applied field direction. This section describes the numerical procedure used to extract $r_{\mathrm{eff}}$ from the phase-field tensor components, implemented in Mathematica.

The zero-field dielectric susceptibility tensor $\chi_{ij}$ is obtained directly from the phase-field output. The optical indicatrix tensor $B_{ij}$ is defined as the matrix inverse of the susceptibility tensor:

$$B_{ij} = \chi_{ij}^{-1}$$

The optical ellipsoid in lab axes $(x,y,z)$ can written as:

$$B_{11}x^{2} + B_{22}y^{2} + B_{33}z^{2} = 1.$$

The semi-axes of this ellipsoid (**Figure S22a**) give the principal refractive indices.

The eigenvalues of the cross-sectional ellipse in the plane containing the beam propagation wave vector $\vec{\boldsymbol{k}}$ yields $n_{o}$ (ordinary) and $n_{e}(\theta)$ (extraordinary), where $\theta$ is the angle between $\vec{\boldsymbol{k}}$ and $z$, the angle made by the propagating beam with $z$ direction.

**Figure S22: a)** a) Index ellipsoid at zero field intersected by the plane normal to the propagating beam direction ($\vec{\boldsymbol{k}}$). The resulting ellipse gives the semi-major axis of $n_{o}$ and a semi-minor axis of $n_{e}(\theta_{film})$. **b)** $xz$-cut of the index ellipsoid showing $n_{e}(\theta_{film})$ indicated by the red points.

The probe beam is incident at 45° to the sample surface normal. Applying Snell’s law at the air–crystal interface,

$$sin \theta_{film} =\frac{sin\left( 45^{\circ} \right)}{n_{o}}$$

gives a refracted angle $\theta_{film} \approx18.65^{\circ}$ inside the material (for $n_{o} = 2.2117$). The refracted wave vector is

$$\vec{\boldsymbol{k}} = (sin \theta_{film}, 0, cos \theta_{film})$$

lying in the lying in the xz-plane (**Figure S22b**). The cross-section of the indicatrix perpendicular to $\vec{\boldsymbol{k}}$ is an ellipse whose semi-axes yield $n_{o}$ and $n_{e}(\theta_{film})$.

For the phase-field susceptibility tensor at zero field ($\chi_{11} = 4.8974, \chi_{22} = 4.8938, \chi_{33} = 4.5997, \chi_{23} = 0.1049, \chi_{13} = -0.0058$), numerical inversion yields $n_{o} = 2.2117$ and $n_{e}(\theta_{film} = 18.65^{\circ}) = 2.2065$.

**Effective electro-optic coefficient**

An electric field $E_{x}$is applied along $x$. The linear EO effect modifies the indicatrix tensor by

$$\Delta B_{ij} = r_{ijk}E_{k}$$

For a field along *x* and the tetragonal/orthorhombic symmetry of KNbO_3_, the non-zero increments are:

$$\Delta B_{11} = r_{111}E_{1},$$

$$\Delta B_{33} = r_{331}E_{1},$$

$$\Delta B_{13} = r_{131}E_{1}.$$

The full modified impermeability tensor $B' = B + \Delta B$ is constructed and the cross-sectional eigenvalue problem is solved again to obtain the field-modified principal indices $n_{o}^{'}$ and $n_{e}^{'}(\theta_{film}).$

Because the applied field $E_{x}$ and the wave vector $\vec{\boldsymbol{k}}$ both lie in the $xz$-plane, the ordinary polarization (along $y$) is decoupled from the electro-optic perturbation in this geometry. Consequently$, n_{o}^{'} = n_{o}$ to first order, while only the extraordinary index changes: $\Delta n_{e}(\theta_{film}) \neq0$. This is confirmed numerically in the simulation.

The effective EO coefficient is defined through the standard relation between the field-induced index change and the zero-field extraordinary index:

$$\Delta n_{e}(\theta_{film}) = -½ r_{\mathrm{eff}} n_{e}^{3}\left( \theta_{film} \right)E_{x}$$

Rearranging,

$$r_{\mathrm{eff}} = -2 \Delta n_{e}(\theta_{film}) / [n_{e}^{3}(\theta_{film}) E_{x}]$$

where $\Delta n_{e}(\theta_{film}) = n_{e}(\theta_{film}) - n_{e}^{'}(\theta_{film})$ and $E_{x}$ is the applied field magnitude. This expression directly converts the phase-field electro-optic tensor components ($r_{13}, r_{33}, r_{51}$) and the zero-field optical indicatrix into the single scalar $r_{\mathrm{eff}}$ that is compared to the experimentally measured lock-in signal.

| Material | $\boldsymbol{r}_{\mathbf{eff}}$ ($\boldsymbol{pm}\boldsymbol{V}^{\boldsymbol{-1}}$) | Thickness (nm) |
| --- | --- | --- |
| BaTiO_3_ on SrTiO_3_/Si | 148 | 130 |
| BaTiO_3_ on SrTiO_3_/Si | 1000 | - |
| BaTiO_3_ on SrTiO_3_/Si | 550 | 150 |
| BaTiO_3_ on SrTiO_3_/Si | 140 | 78 |
| BaTiO_3_ on SrTiO_3_/Si | 46 | 80 |
| BaTiO_3_ on SrTiO_3_/Si | 119 | 100 |
| LiNbO_3_ single crystal | 23 | - |
| KNbO_3_ on SrTiO_3_/Si (Expt., This work) | 113 | 18 |
| KNbO_3_ on SrTiO_3_/Si (Expt., This work) | 91 | 48 |
| KNbO_3_ on SrTiO_3_/Si (Expt., This work) | 146 | 96 |
| KNbO_3_ on SrTiO_3_/Si (Theory, this work) | 88 | 18 |
| KNbO_3_ on SrTiO_3_/Si ($\boldsymbol{\epsilon=-0.8\%}$) (Theory, this work) | 900 | 18 |

**Table S2:** Tabulated film thickness and $r_{\mathrm{eff}}$used in **Figure 5d, Main Text**.

**References**

[1]	D. M. Collins, in *Pressure, Density, and Flux Formulas of Importance to Molecular Beam Epitaxy CRM-346*, Varian Associates Corporate Solid-State Laboratory Inc., Palo Alto, California, **1979**.

[2]	D. G. Schlom, J. S. Harris, *“MBE Growth of High Tc Superconductors,” Molecular Beam Epitaxy:  Applications to Key Materials*, Edited By R.F.C. Farrow (Noyes, Park Ridge), **1995**.

[3]	L. B. Loeb, *The Kinetic Theory of Gases: Being a Text and Reference Book Whose Purpose Is to Combine the Classical Deductions with Recent Experimental Advances in a Convenient Form for Student and Investigator*, 2nd Ed., Pp. 19, 106, McGraw-Hill, **1934**.

[4]	Y. Yang, S. Yin, Q. Yu, Y. Zhu, J. Ding, R. Zhang, C. Ophus, M. Asta, R. O. Ritchie, A. M. Minor, *Nat. Commun.* **2024**, *15*, 1402.

[5]	Z. Wang, B. H. Goodge, D. J. Baek, M. J. Zachman, X. Huang, X. Bai, C. M. Brooks, H. Paik, A. B. Mei, J. D. Brock, J.-P. Maria, L. F. Kourkoutis, D. G. Schlom, *Phys. Rev. Mater.* **2019**, *3*, 073403.

[6]	A. Ross, V. Gopalan, L.-Q. Chen, *ArXiv* **2026**.

[7]	N. Creange, K. P. Kelley, C. Smith, D. Sando, O. Paull, N. Valanoor, S. Somnath, S. Jesse, S. V Kalinin, R. K. Vasudevan, *Mach. Learn. Sci. Technol.* **2021**, *2*, 045002.

[8]	V. Gopalan, R. Raj, *Ferroelectrics* **1997**, *200*, 343.

[9]	T.-H. Lee, D.-H. Kim, B.-Y. Kim, H.-Y. Choi, J.-H. Oh, C.-Y. Kang, S. Nahm, *Acta Mater.* **2016**, *112*, 53.

[10]	J. Lin, J. Cheng, P. Li, W. Chen, H. Huang, *Superlattices Microstruct.* **2019**, *130*, 168.

[11]	W. C. Dunlap, R. L. Watters, *Physical Review* **1953**, *92*, 1396.
